# Epigenetic markers of disease risk and psychotherapy response in anxiety disorders – a longitudinal analysis of the DNA methylome

**DOI:** 10.1038/s41380-025-03038-5

**Published:** 2025-04-25

**Authors:** Katharina Domschke, Miriam A. Schiele, Óscar Crespo Salvador, Lea Zillich, Jan Lipovsek, Andre Pittig, Ingmar Heinig, Isabelle C. Ridderbusch, Benjamin Straube, Jan Richter, Maike Hollandt, Jens Plag, Thomas Fydrich, Katja Koelkebeck, Heike Weber, Ulrike Lueken, Udo Dannlowski, Jürgen Margraf, Silvia Schneider, Elisabeth B. Binder, Andreas Ströhle, Winfried Rief, Tilo Kircher, Paul Pauli, Alfons Hamm, Volker Arolt, Jürgen Hoyer, Hans-Ulrich Wittchen, Angelika Erhardt-Lehmann, Anna Köttgen, Pascal Schlosser, Jürgen Deckert

**Affiliations:** 1 Department of Psychiatry and Psychotherapy, Medical Center - University of Freiburg, Faculty of Medicine, University of Freiburg, Freiburg, Germany; 2 German Center for Mental Health (DZPG), Partner Site Berlin/Potsdam, Berlin, Germany; 3 Institute of Genetic Epidemiology, Faculty of Medicine and Medical Center – University of Freiburg, Freiburg, Germany; 4 Translational Psychotherapy, Institute of Psychology, Georg-August-University of Göttingen, Göttingen, Germany; 5 Institute of Clinical Psychology & Psychotherapy, Technische Universität Dresden, Dresden, Germany; 6 Department of Psychiatry and Psychotherapy & Center for Mind Brain and Behavior - CMBB, Philipps-University Marburg, Marburg, Germany; 7 Department of Psychology, Experimental Psychopathology, University of Hildesheim, Hildesheim, Germany; 8 Department of Psychology, Biological and Clinical Psychology/Psychotherapy, University of Greifswald, Greifswald, Germany; 9 Faculty of Medicine, Institute for Mental Health and Behavioral Medicine, HMU Health and Medical University Potsdam, Potsdam, Germany; 10 Department of Psychology, Faculty of Life Sciences, Humboldt-Universität zu Berlin, Berlin, Germany; 11 LVR-University Hospital, Department of Psychiatry and Psychotherapy, University of Duisburg-Essen, Essen, Germany; 12 Department of Psychiatry, Psychosomatics, and Psychotherapy, Center of Mental Health, University Hospital of Würzburg, Würzburg, Germany; 13 Institute for Translational Psychiatry, University of Muenster, Muenster, Germany; 14 Mental Health Research and Treatment Center, Department of Clinical Psychology and Psychotherapy, Ruhr-Universität Bochum, Bochum, Germany; 15 Mental Health Research and Treatment Center, Department of Clinical Child and Adolescent Psychology, Ruhr-Universität Bochum, Bochum, Germany; 16 Dept. Genes and Environment, Max Planck Institute for Psychiatry, Munich, Germany; 17 Department of Psychiatry and Psychotherapy, Campus Charité Mitte, Charité – Universitätsmedizin Berlin, Berlin, Germany; 18 Department of Clinical Psychology and Psychotherapy & Center for Mind Brain and Behavior - CMBB, Philipps-University Marburg, Marburg, Germany; 19 Department of Psychiatry and Psychotherapy, University of Marburg, Marburg, Germany; 20 Department of Psychology (Biological Psychology Clinical Psychology, and Psychotherapy), Center of Mental Health, University of Würzburg, Würzburg, Germany; 21 Department of Epidemiology, Johns Hopkins University Bloomberg School of Public Health, Baltimore, MD USA; 22 Centre for Integrative Biological Signalling Studies (CIBSS), University of Freiburg, Freiburg, Germany

**Keywords:** Diagnostic markers, Psychiatric disorders

## Abstract

Epigenetic mechanisms such as DNA methylation are hypothesized to play a pivotal role in the pathogenesis of anxiety disorders and to predict as well as relate to treatment response. An epigenome-wide association study (EWAS) (Illumina MethylationEPIC BeadChip) was performed at baseline (BL), post-treatment (POST) and 6-month follow-up (FU) in the so far largest longitudinal sample of patients with anxiety disorders (*N* = 415) treated with exposure-based cognitive behavioral therapy (CBT), and in 315 healthy controls. Independent of comorbidity with depression, anxiety disorders were significantly (*p* ≤ 6.409E–08) associated with altered DNA methylation at 148 CpGs partly mapping to genes previously implicated in processes related to anxiety, brain disorders, learning or plasticity (e.g., *GABBR2*, *GABRD*, *GAST*, *IL12RB2*, *LINC00293*, *LOC101928626*, *MFGE8*, *NOTCH4*, *PTPRN2*, *RIMBP2*, *SPTBN1*) or in a recent cross-anxiety disorders EWAS (*TAOK1*) after pre-processing and quality control (*N* = 378 vs. *N* = 295). Furthermore, BL DNA methylation at seven and three CpGs, respectively, was suggestively (*p* < 1E–5) associated with treatment response at POST (*ABCA7*, *ADRA2C*, *LTBR*, *RPSAP52*, *SH3RF3*, *SLC47A2*, *ZNF251*) and FU (*ADGRD1*, *PRSS58*, *USP47*). Finally, suggestive evidence for dynamic epigenome-wide DNA methylation changes along with CBT response emerged at four CpGs from BL to FU (*ADIPOR2*, *EIF3B*, *OCA2*, *TMCC1*). The identification of epigenetic biomarkers may eventually aid in developing environment-based preventive strategies aimed at increasing resilience by providing deeper molecular insights into the mechanisms underlying anxiety disorders. Defining epigenetic signatures as predictors or key mechanisms in exposure-based interventions could pave the way for more targeted and personalized treatments for anxiety disorders.

## Introduction

Anxiety disorders – constituting the most frequent mental disorders with a 12-month prevalence of 10–14% [1–3] – confer a considerable individual and socioeconomic burden [4]. This is partly due to treatment resistance to first-line pharmacological or cognitive behavioral therapy (CBT) in 20–50% of patients [5–8] contributing to a substantial chronicity, with anxiety disorders ranking 6th among all disorders regarding the years lived with disability (YLDs) worldwide [9] (for review see [10]).

Biomarkers indicating disease risk, predicting treatment outcome or constituting potential mechanistic correlates of treatment response are expected to inform targeted preventive interventions as well as individualized innovative therapeutic approaches and thus might ultimately aid in reducing both incidence and treatment resistance of anxiety disorders [cf. [11, 12]]. As in humans, brain tissue is not accessible in vivo, particularly not in a longitudinal approach, biomarkers measurable in peripheral tissue are urgently warranted. Epigenetic mechanisms – positioned at the interface between genetics and environment – have been suggested to be informative in this regard [13]. Multiple studies have pointed to differential blood DNA methylation patterns to constitute potential biomarkers of anxiety disorder risk or possible mechanistic correlates of response to psychotherapeutic treatment on a candidate gene level [for review see [14–18]].

On a DNA methylome level, however, only five epigenome-wide association studies (EWAS) applying a hypothesis-free approach have investigated peripheral epigenetic disease markers in anxiety disorders in adults so far. Using the Infinium HumanMethylation450 BeadChip, Shimada-Sugimoto et al. [19] identified 40 mostly hypomethylated CpGs in several pathways such as ”positive regulation of lymphocyte activation“ to be significantly associated with panic disorder (PD) in a sample of 48 patients *vs*. 48 healthy controls. Also applying the Infinium HumanMethylation450 BeadChip, Iurato et al. [20] discerned a significant hypermethylation at cg07308824 in an enhancer region of the Homo Sapiens Headcase Homolog (Drosophila) (*HECA*) gene in 49 female PD patients *vs*. 48 healthy controls, which was replicated in an independent sample of 131 cases and 169 controls. In the same cohort, a follow-up analysis on DNA methylation markers of PD in association with cumulative stress-weighted life events revealed a trend for differential methylation in the proximity of the Pyridine Nucleotide-Disulphide Oxidoreductase Domain 1 (*PYROXD1*) and Glucose-Fructose Oxidoreductase Domain Containing 2 (*GFOD2*) genes [21]. A meta-analysis of the two above-mentioned studies [19, 20] comprising a total of 251 participants identified 61, mostly hypomethylated CpGs to be significantly associated with PD, primarily located in or near the *PBK*, *SHOC1*, *TSBP1*, *NDUFAF4*, *CD2AP*, *PIK3C2G*, *SLCO1A2*, *ACSM3*, *ERI2*, *SMARCA5*, *CFAP206*, *MEP1A, CHD2*, *CLASP1, HSPB6* and *SMYD3* gene loci [22]. A third EWAS in PD, based on the Illumina MethylationEPIC BeadChip covering > 90% of the CpGs covered by the HumanMethylation450 BeadChip plus an additional ~350,000 CpGs, yielded suggestive evidence for decreased methylation at cg19917903 in the Cilia and Flagella Associated Protein 46 (*CFAP46*) in 56 patients with PD *vs*. 60 healthy controls [23]. The to date only EWAS in social anxiety disorder based on the Illumina MethylationEPIC BeadChip reported two differentially methylated regions located within the genes coding for the Solute Carrier Family 43 Member 2 (*SLC43A2*) and Tenascin XB (*TNXB*) to be associated with the disorder in a sample of 66 patients and 77 healthy controls [24]. Finally, in 618 patients with mixed anxiety disorders (PD, social anxiety disorder, agoraphobia or generalized anxiety disorder) *vs*. 514 controls, Hettema et al. [25] discerned 280 methylome-wide significant associations in monocytes using MBD-seq, with CpGs located in the Zinc Finger Protein 823 (*ZNF823*) and Fizzy And Cell Division Cycle 20 Related 1 (*FZR1*) genes constituting the most robust hits, and 82 hits in granulocytes.

On a therapy-epigenetic level, to the best of our knowledge only two studies so far have investigated epigenome-wide correlates of treatment-response in PD: Ziegler et al. [23] – applying the Illumina MethylationEPIC BeadChip – observed suggestive evidence for Interleukin 1 Receptor Type 1 (*IL1R1*) methylation to increase along with response to a 6-week CBT in 47 PD patients from baseline to post-treatment. Another study using the Illumina HumanMethylation450 BeadChip failed to discern significantly differential methylation patterns after one exposure session, post 6–8-week CBT or at a 2-month follow-up in 42 PD patients [26]. The top scoring CpGs changing in DNA methylation after exposure or over the complete course of CBT, without, however, relating to clinical response or non-response, were located in the Serotonin 3A Receptor (*HTR3A*), Arginase 1 (*ARG1*), C1D Nuclear Receptor Corepressor (*C1D*) and NudE Neurodevelopment Protein 1 (*NDE1*) genes [26].

In sum, there is burgeoning evidence for peripheral epigenome-wide markers of anxiety disorders or treatment response in anxiety disorders. However, apart from the most recent cross-disorder study by Hettema et al. [25], sample sizes of the presently available studies were small. Also, study designs covered only relatively short observation periods, mostly without follow-up time points. Finally, except from Hettema et al. [25], studies have focused on individual anxiety disorders such as PD despite substantial evidence for a shared genetic and phenotypic architecture across different anxiety disorders [27–32].

Thus, the present cross-disorder EWAS – in the so far largest longitudinal sample of 415 patients with anxiety disorders (panic disorder [PD] with or without agoraphobia, social anxiety disorder, multiple specific phobias) undergoing CBT – aimed at identifying (a) epigenetic disease markers of anxiety disorders by applying a case-control approach, (b) baseline epigenetic markers possibly predictive of treatment response to CBT, and (c) mechanistic correlates of CBT response by assessing DNA methylome changes along with treatment response longitudinally over the course of treatment and at 6-month follow-up.

## Methods

### Patient sample

The presently analyzed sample of 415 patients with anxiety disorders (AD) (238 female, 177 male; mean age ± SD: 33.29 ± 11.3 years; panic disorder [PD] and/or agoraphobia [AG]: *N* = 257; social anxiety disorder [SAD]: *N* = 129; multiple specific phobias [SP]: *N* = 29) comprises a subsample of patients (a) included in a multicenter randomized controlled trial (project P1) on temporally intensified exposure *vs*. standard non-intensified exposure within a standardized cognitive behavioral therapy setting [33, 34] (see “Treatment” below) and (b) with available biodata (blood samples) ascertained within project P5 (“Genetic and epigenetic mechanisms of treatment response”) of the PROTECT‐AD (“Providing Tools for Effective Care and Treatment of Anxiety Disorders“) consortium funded by the German Federal Ministry of Education and Research (BMBF).

Inclusion criteria were a primary diagnosis of PD, AG, SAD, or multiple SP according to DSM-5 criteria, age between 15 and 70 years, baseline severity ≥ 19 on the Hamilton Anxiety Rating Scale (HAM-A) and ≥ 4 on the Clinical Global Impression (CGI) scale, outpatient status, ability to attend sessions, and German language proficiency. Exclusion criteria comprised current comorbid psychotic or substance use disorders except nicotine according to DSM-5, concomitant psychotherapy, acute suicidality or general medical contraindications, and isolated specific phobia. Diagnoses were assessed by experienced clinical psychologists and psychotherapists and ascertained via the computer-administered version of the Composite International Diagnostic Interview (CIDI). Concomitant psychopharmacological treatment was allowed if dosage was stable for at least three months before study inclusion in case of a newly introduced medication and for at least two months in case of dosage adaptation of an existing medication, and medication remained unchanged during the trial [34]. For epigenetic analyses, participants had to be of self-reported European descent. Additional exclusion criteria comprised illegal drug use, severe somatic or neurological disorders, and medication with MAO-inhibitors or valproate. For detailed demographic and clinical characteristics of the final patient sample after data pre-processing and quality control (cf. below) please see Table 1.

**Table 1:** Demographic and clinical characteristics after data pre-processing and quality control.

Case-Control Analysis (T0)			
	Patients	Healthy Controls	*P*-Value
Total	378 (100%)	295 (100%)	
Male	163 (43.1%)	130 (44.1%)	0.78
Female	215 (56.9%)	165 (55.9%)	
Mean Age (Years ± SD)	33.2 ± 11.3	35.2 ± 11.7	0.22
*Main Diagnoses*			
Panic Disorder and/or Agoraphobia	231 (61.1%)	n.a.	
Social Anxiety Disorder	119 (31.5%)	n.a.	
Multiple Specific Phobia	28 (7.4%)	n.a.	
*Comorbidities*			
Generalized Anxiety Disorder	6 (1.6%)	n.a.	
Depression^a^	122 (32.3%)	n.a.	
Obsessive-Compulsive Disorder	1 (0.3%)	n.a.	
Nicotine Dependence	21 (5.6%)	n.a.	
Number of Smokers	99 (26.2%)	not available	
HAM-A Baseline Score	24.48 ± 5.28	n.a.	
SSRI^b^	42 (11.1%)	n.a.	
SNRI^b^	21 (5.6%)	n.a.	
NaSSA^b^	11 (2.9%)	n.a.	
TCA^b^	17 (4.5%)	n.a.	
CBT Response Prediction Analysis			
	**T0–T1**	**T0–T2**	
Total	336 (100%)	306 (100%)	
Male	143 (42.6%)	134 (43.8%)	
Female	193 (57.4%)	172 (56.2%)	
Treatment Responders^c^	170 (50.6%)	200 (65.4%)	
HAM-A Difference Score	−11.58 ± 8.25	−14.42 ± 7.95	
SSRI^b^	35 (10.4%)	30 (9.8%)	
SNRI^b^	18 (5.4%)	14 (4.6%)	
NaSSA^b^	9 (2.7%)	7 (2.3%)	
TCA^b^	16 (4.8%)	15 (4.9%)	
CBT Response Mechanism Analysis			
	**T0–T1**	**T0–T2**	
Total	265 (100%)	226 (100%)	
Male	121 (45.6%)	103 (45.6%)	
Female	144 (54.3%)	123 (54.4%)	
Treatment Responders^c^	133 (50.2%)	141 (62.4%)	
SSRI^b^	28 (10.6%)	21 (9.3%)	
SNRI^b^	13 (4.9%)	12 (5.3%)	
NaSSA^b^	8 (3%)	6 (2.7%)	
TCA^b^	13 (4.9%)	13 (5.8%)	

Sample characteristics are shown for the final samples of patients with anxiety disorders and the matched healthy control group with clinical data and DNA methylation data available after data pre-processing and quality control for the three sub-analyses (see methods section).

*CBT* cognitive behavioral therapy, *T0* baseline (BL), *T1* post-treatment (POST), *T2* 6-month follow-up (FU), *SSRI* selective serotonin re-uptake inhibitors, *SNRI* selective serotonin and norepinephrine re-uptake inhibitors, *NaSSA* noradrenaline and selective serotonin agonists, *TCA* tricyclic antidepressants, *n.a*. not applicable.

^a^depression was defined as diagnosis of major depressive disorder, single episode or recurrent.

^b^psychopharmacological medication had to be stable for at least three months before study inclusion in case of a newly introduced medication and for at least two months in case of dosage adaptation of an existing medication, and remained unmodified during the course of cognitive behavioral therapy (see section Materials and Methods, Patient sample).

^c^responders: ≥50% decrease in Hamilton Anxiety Rating Scale (HAM‐A) score ascertained with the Structured Interview Guide for the Hamilton Anxiety Scale (SIGH‐A) [39]. Between-group comparisons were carried out by Chi-square tests for dimensional data or Students t-tests for categorical data.

The study was performed according to the Declaration of Helsinki and was approved by the Ethics Committee of Technische Universität Dresden, Germany (EK 234062014). The overarching clinical trial was registered (NIMH Protocol Registration System: 01EE1402A and German Register of Clinical Studies: DRKS00008743). Written informed consent was obtained from all participants prior to participation. All methods were performed in accordance with the relevant guidelines and regulations.

### Healthy control sample

A control group of 315 healthy participants of European descent – recruited at the Max-Planck-Institute of Psychiatry (MPIP), Munich, Germany, from a Munich-based community sample [35], and within the framework of the Collaborative Research Centre CRC TRR-58 “Fear, Anxiety, Anxiety Disorders” at the Department of Psychiatry, University of Wuerzburg, Germany [36, 37] – was matched to the patient group by age and sex (frequency matching). Probands with a past or current DSM-IV axis I disorder as ascertained by experienced psychologists (MPIP: Munich Composite International Diagnostic Interview [DIA-X/M-CIDI]; TRR58: Mini International Neuropsychiatric Interview [MINI]), past or current severe neurological or somatic disorders, current intake of centrally active medication, illegal drug use, or pregnancy were excluded [36, 38]. For probands, smoking data was not available. For detailed characteristics of the final healthy control sample after data pre-processing and quality control (cf. below) see Table 1.

Written informed consent was obtained from all probands prior to participation. This part of the study was approved by the ethics committee of the Ludwig-Maximilians-University Munich (project no. 318/00) and of the University of Würzburg (project no. 304/15), Germany, respectively.

### Treatment

All patients received standardized, manualized CBT [33, 34]. Briefly, CBT comprised a total of 14 sessions (100 min. each). Sessions 1–4 were focused on rapport, psychoeducation, functional behavioral analyses, identification of central concerns and maladaptive anxiety control strategies, goal setting, and development of the exposure rationale. Sessions 5–10 comprised therapist-guided in vivo exposure exercises (except session 7), with patient-guided exposure in-between sessions. Sessions 11 and 12 focused on maintenance, relapse prevention, and planning of continued self-guided exposure training. Sessions 13 and 14 served as booster sessions at two and four months post-CBT, respectively. Patients were randomized to receive the same CBT protocol either comprising a temporally intensified exposure (PeEx‐I) phase of 2 weeks (3 exposure sessions per week), resulting in a total of 6 weeks of CBT, or standard non-intensified exposure à one exposure session per week over the course of 10 weeks (PeEx‐S).

The primary outcome measure was based on the Hamilton Anxiety Rating Scale (HAM‐A) as ascertained by the Structured Interview Guide for the Hamilton Anxiety Scale (SIGH‐A) [39] at baseline (BL; T0), post-treatment (POST; T1) and 6-month follow-up (FU; T2). Treatment response was defined as ≥ 50% decrease in HAM‐A score [40]. As in the main clinical study [34], no statistically significant clinical difference regarding treatment effect as measured by HAM-A change from T0 (BL) to T1 (POST) or from T0 (BL) to T2 (FU) could be discerned between the PeEx‐I and the PeEx‐S group. Therefore, no control for ‘treatment group’ was applied in all further analyses.

### Blood sampling

EDTA-blood was taken at T0 (BL), T1 (POST) and T2 (FU). DNA was isolated using the FlexiGene DNA Kit (QIAGEN, Hilden, Germany) and stored at −80 °C until further processing.

### DNA methylation analyses

After bisulfite conversion, DNA methylation at ~865,000 sites was quantified by means of the Infinium MethylationEPIC Kit (Illumina, San Diego, USA). Bisulfite conversion, hybridization and processing were performed according to the manufacturer’s instructions at Life & Brain, Bonn, Germany. Epityping of case and control samples was performed together in the same place and using the same array.

### Quality control and preprocessing of DNA methylation data

The raw methylation data was processed and quality-controlled with a modified version of the CPACOR pipeline [41]. This includes calculation of principal components of the control probes which were used in the analysis to adjust for technical measurement variance. White blood cell type (WBC) composition of each individual was estimated based on 100 CpGs by the Houseman method [42] as implemented in the minfi R package [43]. Fifty-seven samples were identified as outliers and excluded from the analysis based on multiple quality control criteria. These criteria included abnormal staining values in red or green channels, discrepancies between reported and predicted sex, outliers in control probes (BS I-C, BS II, Specificity I, and II) that indicate potential technical issues, and significant batch effects that could introduce systematic biases. Additionally, samples with a call rate below 0.95, indicating unreliable probe detection, and those with poor signal, defined as completely lacking signal in at least 5% of probes, were excluded. After data pre-processing and quality control, DNA methylation data from a final sample of 378 patients and 295 controls was thus available for case-control analysis at BL. For the treatment prediction analysis relating BL methylation to clinical treatment response, both DNA methylation and longitudinal clinical data were available for 336 patients regarding T1, and for 306 patients regarding T2. For the analysis of methylation changes from before (BL) to after treatment (POST) (T0–T1) or at follow-up (FU) (T0–T2), both DNA methylation and longitudinal clinical data were available for 265 and 226 patients, respectively. For detailed demographic and clinical characteristics of the final samples at all timepoints see Table 1.

### Epigenome-wide association analyses

As described previously [23, 44], EWAS was performed in two steps. First, technical variance was adjusted for in terms of the first ten principal components of the control probes and the estimated WBC type composition (CD8 T cells, CD4 T cells, natural killer cells, B-cells, monocytes, granulocytes). The resulting residuals from the M values of the DNA methylation (which are a logarithmic transformation of β-values) were then used in the second analysis step, which differs for the three types of analyses conducted here:

1. For the case-control EWAS in anxiety disorder patients *vs*. healthy controls, adjusted methylation levels at BL were compared between patients and controls by means of a non-paired Welch test, which allows for different group sizes as well as different variances within each group, and was chosen to make use of the complete control group. As cases and controls were balanced for age and sex (see Table 1), no adjustment was deemed necessary. The potential impact of comorbid depression (defined as comorbid depressive episode or recurrent major depressive disorder) was analyzed *post hoc* for all CpGs significantly associated with anxiety disorders. For each of those CpGs, a linear model was fitted with the adjusted methylation levels as outcome and group (patients *vs*. controls) and comorbid depression (yes *vs*. no) as binary predictors. Information on smoking was not available for the control group and thus could not be accounted for a priori, but was considered *post hoc* against an available EWAS on the influence of smoking on blood DNA methylation [45]. In addition, an EWAS of smoking status was performed in the patient group (*N* = 378), and the overlap with CpG sites identified in the case-control EWAS was tested. Here, a robust linear regression was performed based on beta values, controlling for sex, age, cell type composition and the principal components of the control probes. To investigate the broader biological processes the significant CpG sites are involved in, a Gene Ontology (GO) enrichment analysis was performed for biological processes on the gene level using clusterProfiler [46].
2. For the treatment response prediction analyses in anxiety disorder patients, logistic regression models were used to determine association of BL methylation levels with clinical response at T1 (POST) or T2 (FU) as the binary outcome. For efficiency, both steps of adjustment for technical variance and the association step were combined in the same model. In addition to technical variance, models were adjusted for the following variables: age, sex, smoking status, comorbid depression, and the HAM-A score at BL. In addition, continuous analyses of the percentage of HAM-A score change from BL to T1 and from BL to T2 were performed as supplementary analyses. These models were adjusted for the same covariates with the exception of the HAM-A score at BL.
3. The longitudinal pre-post analyses compared BL methylation levels with methylation levels at a T1 or T2 stratified for responder status in anxiety disorder patients. Differences in adjusted methylation levels were calculated within each patient and then analyzed with a one-sample t-test. Since baseline characteristics, including psychiatric medication, were kept unchanged within each person for the duration of therapy, no further adjustment was applied.

Prior to analyses, 16,884 CpGs were removed from the data due to overlapping with single nucleotide polymorphisms (SNP) (distance to SNP ≤ 5 base pairs [bp] and European ancestry based minor allele frequency ≥ 0.01). In addition, 48,507 CpGs potentially affected by cross-hybridisation, i.e. where a CpG probe binds to multiple loci in the genome, were removed (https://github.com/sirselim/illumina450k_filtering) [47, 48]. For all analyses, the threshold for statistical significance was set to *p* ≤ 6.409E–08, corresponding to a Bonferroni correction for the 780,145 evaluated CpGs (0.05/780,145). Associations at a significance level of *p* < 1E–5 were considered suggestive. The genomic inflation factor lambda [49] was calculated, and QQ-plots (see Supplementary Fig. S1 for the case-control analysis) were inspected visually.

## Results

### Case-control association analysis

**Fig. 1:**
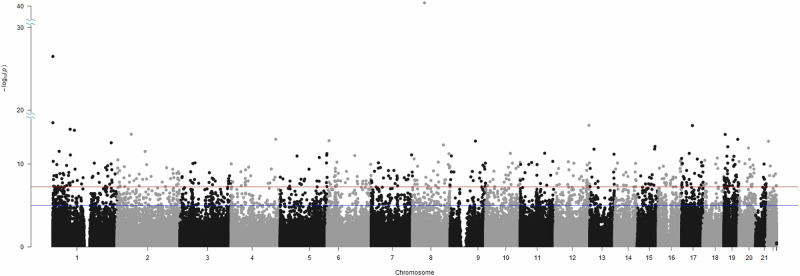
Manhattan plot of the epigenome-wide association study (EWAS) in patients with anxiety disorders (*N* = 378) *vs*. healthy controls (*N* = 295). The x axis shows the chromosomal position, the y axis shows *p*-values of the case-control analysis on a -log_10_ scale. The upper (red) horizontal line indicates the threshold for significant sites (*p* = 6.409E–08), the lower (blue) horizontal line indicates the threshold for suggestive sites (*p* = 1E–5).

The present epigenome-wide case-control association study in the final sample of 378 anxiety disorder patients *vs*. 295 healthy controls revealed significantly differential methylation at 495 CpGs (see Fig. 1). A *post hoc* analysis adjusted for comorbidity with depression revealed that 148 of those 495 CpGs remained significant for association with anxiety disorders (see Table 2), and 324 CpGs were still above the threshold for suggestive significance, while 23 CpGs were neither significantly nor suggestively associated with anxiety disorders any longer (for both see Supplementary Table S1). When cross-referencing the presently identified significant hits with the 4496 CpGs reported to be associated with smoking in a recent EWAS [45], no overlaps could be identified. While 88 epigenome-wide significant CpG sites were observed in the EWAS of smoking status in the patient group (*N* = 378) at baseline (see also Supplementary Table S2), none of them overlapped with the 495 CpG sites associated with case control status. GO enrichment analysis revealed biological processes related to myotube differentiation, the cell cycle and the gamma-aminobutyric acid signaling pathway (see also Supplementary Table S3), although no GO term remained significant after multiple testing correction.

**Table 2:** Epigenome-wide significantly differentially methylated CpG sites (*p* ≤ 6.409e–08) in patients with anxiety disorders (*N* = 378) vs. healthy controls (*N* = 295).

CpG	T-Value	*P*-Value	Mean of Patients - Controls (Δβ)	Nearest Gene Annotation
cg16581032	14.39	4.08e–41	0.018 (11.22%)	*LINC00293*
cg22519184	11.6	3.17e–27	0.099 (28.63%)	*LOC101928626*
cg26679879	8.33	1.06e–15	0.069 (23.62%)	*LOC101928626*
cg24390820	−8.13	2.16e–15	−0.007 (−0.93%)	*RIMBP2*
cg00689014	−8.13	2.35e–15	−0.013 (−2.2%)	*GAST*
cg02339888	−7.98	6.54e–15	−0.017 (−3.18%)	*IL12RB2*
cg26077214	−7.94	8.64e–15	−0.021 (−3.35%)	*LINC01725*
cg03600868	−7.8	2.60e–14	−0.019 (−2.68%)	*SPTBN1*
cg16636182	−7.79	2.70e–14	−0.008 (−0.87%)	*KDM4B*
cg15403961	−7.61	1.05e–13	−0.023 (−3.9%)	*GALNTL6*
cg26325497	−7.61	1.06e–13	−0.017 (−4.44%)	*MIR519B*
cg06567972	−7.55	1.52e–13	−0.01 (−1.64%)	*SNRNP48*
cg03470837	−7.53	1.72e–13	−0.016 (−2.67%)	*GABBR2*
cg09414262	−7.52	1.83e–13	−0.015 (−1.91%)	*LINC00895*
cg25746764	7.47	2.76e–13	0.004 (1.2%)	*ACBD3*
cg02969418	−7.38	5.05e–13	−0.014 (−2.02%)	*TAF2*
cg11992550	−7.33	7.69e–13	−0.017 (−2.67%)	*MFGE8*
cg23849483	−7.31	8.29e–13	−0.014 (−2.11%)	*SYDE1*
cg01597342	−7.26	1.18e–12	−0.019 (−2.77%)	*DLGAP4-AS1*
cg01706621	−7.22	1.55e–12	−0.017 (−2.35%)	*LINC00052*
cg06531158	−7.21	1.63e–12	−0.001 (−0.08%)	*LINC00445*
cg10013501	−7.12	2.95e–12	−0.004 (−0.46%)	*RUNX3*
cg27233071	−7.12	3.07e–12	−0.026 (−4.86%)	*SULT1C2P1*
cg04646695	−7.04	5.00e–12	−0.016 (−2.65%)	*IZUMO1R*
cg25213418	−7.03	5.23e–12	−0.014 (−2.04%)	*TAOK1*
cg11415852	−7.03	5.30e–12	−0.012 (−1.46%)	*SORBS1*
cg16559448	−7.02	5.64e–12	−0.019 (−3.03%)	*CNOT6*
cg07437919	−7.03	5.74e–12	0.001 (0.18%)	*SLC45A4*
cg14001871	−7.01	6.56e–12	−0.006 (−0.72%)	*ATP11A*
cg10508217	−6.97	8.10e–12	−0.005 (−0.7%)	*GNG12-AS1*
cg15609150	−6.96	8.73e–12	−0.019 (−3.3%)	*MAML1*
cg00449068	−6.95	9.57e–12	−0.017 (−2.41%)	*BEND3*
cg00590386	−6.93	1.06e–11	−0.007 (−1.49%)	*RIC1*
cg00335957	−6.93	1.10e–11	−0.01 (−1.54%)	*CWC27*
cg22565389	−6.92	1.14e–11	−0.002 (−0.33%)	*CNN1*
cg22887498	−6.91	1.17e–11	−0.005 (−0.57%)	*CCER2*
cg15817341	−6.86	1.62e–11	−0.015 (−1.94%)	*ANXA6*
cg14264457	−6.84	1.88e–11	−0.007 (−0.89%)	*CCDC68*
cg12009816	−6.82	2.11e–11	−0.014 (−1.77%)	*RPH3AL*
cg01523759	−6.81	2.22e–11	−0.003 (−0.36%)	*ACSF3*
cg08580015	−6.8	2.40e–11	−0.001 (−0.15%)	*MCPH1*
cg05869737	−6.79	2.52e–11	−0.015 (−2.58%)	*APPBP2*
cg22865640	−6.78	2.73e–11	−0.006 (−1.05%)	*MAFB*
cg20324516	−6.76	3.16e–11	−0.018 (−2.37%)	*LINC01250*
cg26529712	−6.76	3.20e–11	−0.015 (−2.79%)	*LINC01838*
cg19763069	−6.74	3.45e–11	−0.01 (−1.38%)	*LOC101928737*
cg26420606	−6.74	3.50e–11	−0.006 (−0.75%)	*L3MBTL4*
cg03513682	−6.71	4.16e–11	−0.007 (−0.93%)	*GSE1*
cg26903053	−6.69	4.78e–11	−0.003 (−0.34%)	*GABRD*
cg15229773	−6.69	4.82e–11	−0.023 (−3.27%)	*PATE2*
cg15339796	−6.69	5.01e–11	−0.016 (−2.2%)	*TRIB3*
cg01739401	−6.68	5.31e–11	−0.017 (−2.37%)	*ATF1*
cg04258637	−6.66	5.96e–11	−0.026 (−4.95%)	*COX6C*
cg09754810	−6.65	6.04e–11	−0.013 (−1.75%)	*ZFAND3*
cg20191924	−6.64	6.54e–11	−0.012 (−1.54%)	*ACCS*
cg01712359	−6.63	6.93e–11	−0.003 (−0.32%)	*PIEZO1*
cg07320742	−6.63	7.12e–11	−0.016 (−2.28%)	*TAX1BP1*
cg20750930	−6.63	7.21e–11	−0.003 (−0.4%)	*FBRSL1*
cg00986191	−6.63	7.28e–11	−0.01 (−1.22%)	*GRM6*
cg10636163	−6.63	7.29e–11	−0.018 (−2.35%)	*C3orf67*
cg03916902	6.63	7.58e–11	0.004 (2.46%)	*AP4S1*
cg11277380	−6.62	7.75e–11	−0.012 (−1.61%)	*CCDC105*
cg21242417	−6.61	7.81e–11	−0.009 (−1.39%)	*EXD3*
cg15610777	−6.61	7.95e–11	−0.005 (−0.6%)	*CALHM3*
cg15604046	−6.61	8.09e–11	−0.018 (−2.97%)	*TRHDE*
cg22404741	−6.61	8.41e–11	−0.014 (−1.97%)	*LINC02285*
cg26134615	−6.6	8.69e–11	−0.005 (−0.97%)	*OR1A1*
cg00777895	6.58	9.89e–11	0.016 (4.38%)	*SRP9*
cg17272899	−6.57	1.04e–10	−0.006 (−0.78%)	*CTIF*
cg11598427	−6.56	1.11e–10	−0.005 (−0.66%)	*SMOC2*
cg11450546	−6.56	1.13e–10	−0.006 (−0.97%)	*LYPD3*
cg22012693	−6.55	1.15e–10	−0.019 (−2.87%)	*COL22A1*
cg18096895	−6.55	1.16e–10	−0.005 (−0.66%)	*BATF2*
cg20979128	−6.55	1.17e–10	−0.005 (−0.52%)	*ZBTB17*
cg02120071	−6.55	1.17e–10	−0.003 (−0.31%)	*LOC100499194*
cg16311883	−6.54	1.31e–10	−0.019 (−3.46%)	*RFLNA*
cg05348295	6.52	1.37e–10	0.007 (8.41%)	*FAM129B*
cg22513166	−6.51	1.64e–10	−0.015 (−4.55%)	*PPP2R5A*
cg26921458	−6.5	1.71e–10	−0.007 (−0.95%)	*SEPT9*
cg03019505	−6.49	1.72e–10	−0.014 (−2.87%)	*TFIP11*
cg02932021	−6.48	1.84e–10	−0.01 (−1.46%)	*TMC5*
cg02944953	−6.47	1.92e–10	−0.009 (−1.13%)	*SIPA1L3*
cg25372841	−6.47	1.93e–10	−0.005 (−0.68%)	*CYHR1*
cg02655549	−6.46	2.08e–10	−0.01 (−2.18%)	*GPN1*
cg18177613	−6.46	2.11e–10	−0.009 (−1.23%)	*CFAP77*
cg21250048	−6.44	2.39e–10	−0.021 (−3.01%)	*PTEN*
cg08314795	−6.44	2.46e–10	−0.016 (−2.15%)	*LINC01728*
cg20680284	−6.42	2.67e–10	−0.01 (−1.59%)	*LOC401324*
cg15695045	−6.42	2.74e–10	−0.005 (−1.09%)	*PLEKHA6*
cg24172570	−6.39	3.11e–10	−0.013 (−1.65%)	*HIBADH*
cg10117405	−6.4	3.15e–10	0 (−0.02%)	*SRL*
cg14224170	−6.38	3.30e–10	−0.016 (−2.33%)	*SAFB2*
cg02955100	−6.38	3.31e–10	−0.007 (−1.02%)	*PPT2-EGFL8*
cg07132926	−6.38	3.40e–10	−0.021 (−3.02%)	*XPO4*
cg09194742	−6.38	3.40e–10	−0.01 (−1.35%)	*HSPA12A*
cg06516751	−6.38	3.43e–10	−0.022 (−3.79%)	*WDR7*
cg25239226	−6.37	3.49e–10	−0.008 (−0.96%)	*MLYCD*
cg04576317	−6.35	3.98e–10	−0.007 (−0.96%)	*DCAF11*
cg19613624	−6.35	4.01e–10	−0.005 (−0.71%)	*PMEPA1*
cg04976850	−6.35	4.17e–10	−0.015 (−2.44%)	*CLCA2*
cg20618167	−6.34	4.25e–10	−0.001 (−0.09%)	*LOC101928103*
cg14468497	−6.29	5.95e–10	−0.014 (−2.55%)	*RNF112*
cg02294570	−6.29	5.96e–10	−0.013 (−2.45%)	*WDR66*
cg03230491	−6.28	6.08e–10	−0.008 (−1.05%)	*CC2D1A*
cg07790939	−6.25	7.44e–10	−0.012 (−1.45%)	*SCIN*
cg03057072	−6.25	7.44e–10	−0.005 (−0.72%)	*ARHGAP12*
cg01220720	−6.25	7.59e–10	−0.008 (−1.1%)	*NOTCH4*
cg13548652	−6.24	7.84e–10	−0.021 (−2.94%)	*ZCCHC6*
cg21740507	−6.23	8.67e–10	−0.015 (−2.18%)	*IQCA1*
cg20569791	−6.22	8.79e–10	−0.009 (−1.47%)	*PLGRKT*
cg22958188	−6.22	9.32e–10	−0.004 (−0.4%)	*LINC02337*
cg26173773	−6.21	9.39e–10	−0.006 (−0.83%)	*PTPRN2*
cg01216201	−6.21	9.57e–10	−0.006 (−0.73%)	*CCDC57*
cg12213699	−6.2	1.01e–09	−0.015 (−1.89%)	*SYNE2*
cg10988013	−6.19	1.06e–09	−0.015 (−2.12%)	*PAPPA*
cg01137782	−6.19	1.11e–09	−0.008 (−1.06%)	*TIGIT*
cg02641865	−6.16	1.34e–09	−0.02 (−2.79%)	*RLF*
cg19609713	−6.15	1.34e–09	−0.008 (−0.9%)	*CD27-AS1*
cg08857906	6.14	1.44e–09	0.007 (1.64%)	*PPP1R8*
cg02395863	−6.13	1.59e–09	−0.003 (−0.33%)	*RGS7*
cg23131559	−6.1	1.76e–09	−0.008 (−1.18%)	*GDPD3*
cg16933892	−6.09	1.92e–09	−0.006 (−0.88%)	*PI3*
cg22486192	−6.09	1.94e–09	−0.017 (−2.52%)	*MVP*
cg25947600	−6.08	2.06e–09	−0.009 (−1.22%)	*C19orf68*
cg05810476	−6.06	2.28e–09	−0.013 (−2.43%)	*PTEN*
cg16190127	−6.06	2.40e–09	−0.009 (−1.3%)	*WDR27*
cg11748170	−6.06	2.47e–09	−0.01 (−1.21%)	*NOTCH4*
cg02482718	−6.05	2.58e–09	−0.007 (−1.16%)	*AJAP1*
cg13786513	−6.04	2.64e–09	−0.002 (−0.22%)	*PPP1R37*
cg12165864	−6.03	2.70e–09	−0.008 (−0.92%)	*LOC644794*
cg07367519	−6.02	2.83e–09	−0.002 (−0.22%)	*CACNA1I*
cg13486309	−6.02	2.99e–09	−0.015 (−2.12%)	*ASH1L*
cg09143779	−6.01	3.15e–09	−0.01 (−1.41%)	*DSE*
cg08314899	−006	3.21e–09	−0.013 (−1.77%)	*MYBPC1*
cg19118037	−006	3.25e–09	−0.007 (−1.02%)	*AIPL1*
cg01217923	−5.93	5.12e–09	−0.003 (−0.47%)	*ZNF564*
cg03463293	−5.93	5.14e–09	−0.006 (−0.76%)	*C15orf39*
cg25612428	−5.89	6.16e–09	−0.001 (−0.12%)	*ANKRD33B*
cg26222012	−5.88	6.82e–09	−0.001 (−0.15%)	*PRSS37*
cg12660062	−5.85	7.79e–09	−0.003 (−0.49%)	*LOC101926942*
cg15999796	−5.79	1.13e–08	−0.012 (−1.86%)	*MOG*
cg11162741	−5.78	1.19e–08	−0.015 (−2.21%)	*TRAF1*
cg17736252	−5.76	1.34e–08	−0.005 (−0.62%)	*MIGA1*
cg14500563	−5.68	1.99e–08	−0.007 (−0.84%)	*MIR602*
cg05309750	−5.67	2.13e–08	−0.011 (−1.48%)	*GXYLT2*
cg23863528	−5.67	2.19e–08	−0.011 (−1.52%)	*GAB1*
cg14775469	−5.58	3.49e–08	−0.016 (−2.49%)	*SFSWAP*
cg11617879	−5.58	3.60e–08	−0.017 (−2.29%)	*USP22*

CpGs remaining to be significantly associated with anxiety disorders at *p* ≤ 6.409e–08 after statistical adjustment for comorbidity with depression (defined as diagnosis of major depressive disorder, single episode or recurrent); Δβ: Positive values indicate methylation in anxiety disorder patients > methylation in healthy controls, negative values indicate methylation in anxiety disorder patients < methylation in healthy controls.

### Treatment response prediction analysis

Treatment response at POST and FU was suggestively associated with methylation status at seven and three CpGs, respectively (Table 3). There was no overlap between CpGs differentially methylated between patients with anxiety disorders and healthy probands in the case-control EWAS (see above) and those related to treatment response mechanisms (see below). Treatment response as a continuous score, representing the percentage of improvement in HAM-A, was associated with six CpGs at POST and thirteen CpGs at FU (see also Supplementary Table S4). All CpG sites identified in the binary analysis were significant in the continuous analysis (all *p* ≤ 0.0082).

**Table 3:** Epigenome-wide DNA methylation at baseline associated with response to cognitive-behavioral therapy (CBT) at T1 (post-treatment) and at T2 (6-month follow-up) with suggestive significance (*p* ≤ 1E–5) in patients with anxiety disorders (T1: *N* = 336; T2: *N* = 306).

T1							
CpG	Coefficient^a^	SE	Z-Value	*P*-Value ^b^	Nearest Gene Annotation	Previous Functional Evidence	References
cg14814319	−4.685	0.969	−4.834	1.336E–06	*RPSAP52*	Associated with anxiety disorders in B-cells in a cross-anxiety disorder EWAS	[25]
cg10700019	4.548	0.952	4.776	1.791E–06	*ZNF251*	–	–
cg06730721	6.040	1.266	4.770	1.838E–06	*ABCA7*	No involvement in anxiety in *Abca7* KO mice, but implicated in synaptic plasticity and cognition, as potentially relevant for CBT	[91, 92]
cg17982539	3.801	0.805	4.724	2.312E–06	*LTBR*	–	–
cg02325313	−2.662	0.585	−4.553	5.295E–06	*ADRA2C*	*Adra2c* mRNA levels were modulated by stress in a mouse model	[93]
cg01461856	−2.140	0.478	−4.472	7.737E–06	*SLC47A2*	Associated with anxiety disorders in granulocytes and B-cells in a cross-anxiety disorder EWAS	[25]
cg11888727	−4.062	0.909	−4.467	7.951E–06	*SH3RF3*	Associated with anxiety disorders in granulocytes in a cross-anxiety disorder EWAS	[25]
T2							
cg12069641	1.659	0.366	4.528	5.949E–06	*USP47*	–	–
cg00032490	−1.892	0.424	−4.465	8.012E–06	*ADGRD1*	–	–
cg04793888	4.040	0.911	4.435	9.216E–06	*PRSS58*	–	–

Response to CBT was defined according to a T0–T1 or T0–T2 change of ≥ 50% in Hamilton Anxiety Rating Scale (HAM-A) scores (see “Treatment”).

^a^a positive coefficient indicates higher methylation to predict increased response to CBT and vice versa.

^b^adjusted for absolute Hamilton Anxiety Rating Scale (HAM-A) score at T0, technical variance, age, sex, smoking status and comorbidity with depression.

### Treatment response mechanism analysis

A longitudinal analysis of changes in epigenome-wide DNA methylation along with response to CBT did not yield any evidence for dynamic alterations in methylation patterns from BL to POST, but returned suggestive evidence for changes at four CpGs from BL to FU (Table 4). There was no overlap between CpGs displaying dynamic methylation along with treatment response and those differentially methylated between anxiety disorder patients and healthy probands in the case-control EWAS or those related to treatment response prediction (see above).

**Table 4:** Epigenome-wide suggestive (*p* < 1E–5) DNA methylation changes (Mean Diff.) from T0 (baseline) to T1 (post-treatment) and to T2 (6-month follow-up), respectively, in ^a^ responders to cognitive-behavioral therapy (CBT) in patients with anxiety disorders (T0–T1: *N* = 133; T0–T2: *N* = 141).

T0–T1						
CpG	T-Value	*P*-Value	Mean Diff. T0–T1	Nearest Gene Annotation	Previous Functional Evidence	Reference
–	–	–	–	–	–	–
T0–T2						
CpG	T-Value	*P*-Value	Mean Diff. T0-T2	Nearest Gene Annotation	Previous Functional Evidence	Reference
cg04624514	−4.739	5.212E–06	−0.00085 (−0.1%)	*TMCC1*	• CircTmcc1 contributed to the secretion of proinflammatory mediators and glutamate metabolism in astrocytes and subsequently modulated an improvement in spatial memory by mediating neuronal synaptic plasticity • Associated with anxiety disorders in granulocytes in a cross-anxiety disorder EWAS	[25, 94]
cg00666772	4.659	7.317E–06	−0.00018 (−0.02%)	*OCA2*	• Associated with anxiety disorders in monocytes in a cross-anxiety disorder EWAS	[25]
cg21601852	4.612	8.917E–06	−0.0011 (0.12%)	*ADIPOR2*	• Adiponectin-deficient mice exhibited normal contextual fear conditioning but displayed slower extinction learning • Infusions of adiponectin into the dentate gyrus of the hippocampus in fear-conditioned mice facilitated extinction of contextual fear • Targeting adiponectin/AdipoR2 have been suggested to strengthen extinction-based exposure therapies • Adiponectin-KO mice displayed increased anxiety at 9 and 18 months • Associated with anxiety disorders in granulocytes in a cross-anxiety disorder EWAS	[25, 95–97]
cg21474639	4.597	9.472E–06	−0.00041 (−0.05%)	*EIF3B*	–	–

Mean Diff.: negative values indicate a decrease in methylation from T0–T2.

^a^responders to CBT were defined according to a T0 to T1/T2 change of ≥ 50% in Hamilton Anxiety Rating Scale (HAM-A) scores (see “Treatment”).

## Discussion

The present cross-disorder case-control EWAS provided significant evidence for differential DNA methylation associated with anxiety disorders, independent of comorbid depressive symptomatology. Additionally, suggestive associations emerged for DNA methylation patterns potentiall predicting or mediating treatment response to cognitive behavioral therapy (CBT) directly after treatment or at 6-month follow-up in the to date largest sample of patients with anxiety disorder in this context.

### Case-control association: potential neurobiological implications

The presently applied hypothesis-free EWAS approach in anxiety disorder patients revealed significant evidence for epigenetic candidates to be involved in the mediation of disease status, with mostly relative hypomethylation in patients as compared to controls. The top hit maps closest to a gene coding for the Long Intergenic Non-Protein Coding RNA 293 (*LINC00293*), which has been found to be upregulated in multiple sclerosis [50], thereby suggesting a potential role of autoimmune and inflammatory processes also in anxiety disorders [51]. Another two top hits are located in the chromosomal region 1p36.33 near the lncRNA *LOC101928626* gene locus. *LOC101928626* is transcribed as part of a pseudogene of *Septin14*, which encodes a GTP-binding protein involved in the migration of cortical neurons during development [52]. However, *LOC101928626* itself has not yet been functionally characterized to our knowledge. Other exemplarily selected genes or systems displaying significantly altered DNA methylation patterns in the present analysis have previously been associated with anxiety-related phenotypes or neuronal development: For instance, one of the present top hits implicates the *RIMBP2* gene in anxiety disorders, which is in line with a recent genome-wide association study (GWAS) reporting a *RIMBP2* SNP to be associated with anxiety-related bruxism [53]. Furthermore, Gastrin (*GAST*), structurally related to cholecystokinin(CCK)-4, is expressed on mRNA level in human and rat brain systems conferring defensive reactivity including the amygdala, the prefrontal cortex and the locus coeruleus (http://biogps.org; as consulted online on February 27th, 2024) and displays strong affinity to the brain-predominant CCK-B/CCK-2 receptor [54]. Since gastrin-releasing peptide in the basolateral amygdala has been implied in conditioned fear [55], and CCK-4 and its synthetic analogue tetragastrin have been shown to potently induce panic attacks via the CCK-B/CCK-2 receptor [56], it could be speculated that, in analogy, the presently identified decreased methylation at the *GAST* gene, potentially increasing gene expression, might be involved in anxiety disorder pathogenesis as well. The finding of altered methylation at the Interleukin 12 Receptor Subunit Beta 2 (*IL12RB2*) and the Interleukin 12 receptor, beta 2 subunit (*IL12RB2*) locus is well in line with an involvement of pro-inflammatory cytokines in anxiety in general [57] as well as a recent finding of interleukin-12 in particular to be negatively correlated with cortisol levels in PD patients [58]. Spectrin Beta, Non-Erythrocytic 1 (*SPTBN1*) has previously been implied in neural development and function, particularly cortical organization, developmental delay and behavioral deficits [59]. Furthermore, differential methylation has been identified at CpGs mapping to the GABA Type B Receptor Subunit 2 (*GABBR2*) and GABA Receptor Subunit Delta (*GABRD*) belonging to the gamma-aminobutyric acid (GABA) system, which is known to be centrally involved in the pathogenesis of fear, anxiety and anxiety disorders [60–62]. The Milk Fat Globule EGF and Factor V/VIII Domain Containing (*MFGE8*), which is related to microglial activation, has been discerned to be differentially expressed in rat models exhibiting large differences in internalizing and externalizing behavior [63]. The *PTPRN2* gene coding for the Protein Tyrosine Phosphatase Receptor Type N2 has been found to be associated with increased Hospital Anxiety and Depression Scale – Anxiety (HADS-A) scores in a recent GWAS [64]. Finally, two CpGs presently identified to be differentially methylated in anxiety disorders map to the Neurogenic Locus Notch Homolog Protein 4 (*NOTCH4*) gene, which has been identified as a risk factor in schizophrenia [65], but has not been implicated in anxiety-related phenotypes before. However, the fact that glucocorticoid signaling has been shown to activate *Notch4* transcription [66] suggests a potential relevance in the pathogenesis of stress-related disorders such as anxiety disorders as well.

Importantly, the TAO Kinase 1 (*TAOK1*) gene – encoding a serine-threonine kinase previously associated with autism spectrum disorder and neurodevelopmental delay [67] – implied by one of the present top 25 hits was also among the significantly associated genes emerging from a recent cross-anxiety disorder EWAS in monocytes [25]. When not only considering significant results but also findings on a suggestive or lesser significance level as emerging from this cell-type specific EWAS [25], several genes overlapping with the presently identified ones could be discerned (see Supplementary Table S5). Results from other available EWAS in anxiety disorders as detailed in the introduction [19–21, 23] could not be replicated in the present analysis, which might be due the smaller and thus potentially underpowered sample sizes of those previous studies.

In sum, the present EWAS revealed several novel and supported a previous epigenetic candidate potentially involved in the pathophysiology of anxiety disorders, which are to be further explored in future functional analyses. As the present case-control study applied a cross-sectional approach, it remains to be elucidated whether the identified differential methylation patterns are actually causally related to anxiety disorder pathogenesis or rather a consequence of disease status.

### Treatment response prediction and mechanisms: potential neurobiological implications

Epigenome-wide suggestive evidence emerged for DNA methylation patterns at baseline as potential indicators of favorable treatment response to CBT at post-treatment or at 6-month follow-up. As detailed in Table 3, associated CpGs map to genes that in part have previously been found to be involved in synaptic plasticity, cognition as well as the regulation of stress, which might be relevant for successful fear extinction processes and thus the prediction of treatment response in anxiety disorders as well. Interestingly, the *RPSAP52*, *SLC47A2*, *SH3RF3* genes have also been implied in a previous cross-anxiety disorder EWAS [25].

The present longitudinal EWAS revealed suggestive evidence for temporally dynamic DNA methylation patterns potentially underlying treatment response to CBT. In anxiety disorder patients responding to CBT, changes in DNA methylation from baseline to 6-month follow-up were observed at CpGs partly mapping to loci of genes previously implicated in processes related to anxiety, contextual fear conditioning or neuronal synaptic plasticity. The *TMCC1*, *OCA2* and *ADIPOR2* genes have also been identified in a recent cross-anxiety disorder EWAS [25] (for details see Table 4). The observed dynamics in DNA methylation patterns along with response to CBT extend the emerging body of evidence for epigenetic mechanisms possibly constituting biological correlates of psychotherapeutic interventions by complementing previous candidate-gene based studies [18, 68–71].

While the clinical validity of the identified CpGs is currently limited, given that their functional relevance remains unclear and replication is warranted, these findings could in the future contribute to more personalized treatment approaches. If validated, epigenetic biomarkers, such as methylation risk scores, could aid in identifying patients who are more or less likely to benefit from specific psychotherapeutic interventions, and inform adjunctive treatment strategies, such as pharmacological augmentation tailored to an individual’s epigenetic profile. Furthermore, the present findings might inform the development of innovative pharmacological compounds, with for instance further support from the current hits within the gamma-aminobutyric acid signaling pathway for recent efforts in testing α1 sparing, α2/3/5-selective GABAA-receptor positive allosteric modulators in generalized anxiety disorder and panic disorder [72]. While these applications are not yet clinically actionable, they open up novel avenues towards translating epigenetic findings into clinical practice.

The lack of overlap between hits across the three analyses (case-control, prediction, mechanism) might be due to methodological limitations such as smaller sample sizes of the group of patients with available longitudinal data or could be interpreted in the light of different mechanisms underlying development vs. maintenance vs. treatment response of anxiety disorders potentially reflected by distinct epigenetic signatures, respectively [12]. The “Biomarkers, EndpointS, and other Tools” Resource (BEST) of the FDA-NIH Biomarker Working Group [73] clearly distinguishes between ‘susceptibility biomarkers’, aiming at estimating the likelihood of developing an illness, ‘diagnostic biomarkers’, used to detect or confirm presence of a disease and adding a biological perspective to conventional clinical assessment, ‘predictive biomarkers’, aiming at estimating the likelihood of experiencing a therapeutic effect, and ‘monitoring biomarkers’ measured repeatedly for assessing status of a disease or for evidence of effect of a treatment. These biomarkers can be different in terms of their biological function and clinical application. The CpGs presently identified to be differentially methylated between cases and controls have to be primarily considered ‘diagnostic biomarkers’, while the identification of ‘susceptibility biomarkers’ needs to be based on longitudinal data including a time period before disease incidence. The mechanisms of disease in anxiety disorders relating to e.g. the glutamate, serotonin, norepinephrine and GABA systems, with the latter also being implicated in the pathway analysis, in the sense of ‘diagnostic biomarkers’ are most probably distinct from molecular predictors of psychotherapy treatment response to CBT, which might rather be linked to neurotransmitter systems and pathways involved in learning and memory and intracellular mechanisms of synaptic plasticity. With respect to the presently observed dynamic changes in methylation along with treatment response, these should indeed be identical to the identified ‘diagnostic biomarkers’ if to be considered ‘monitoring biomarkers’ relating to the disease status and course. If considered epigenetic mechanisms of action of a psychotherapeutic intervention as in the present case, however, they do not necessarily have to overlap with the identified ‘diagnostic biomarkers’ linked to anxiety per se. Mechanisms of change underlying an effective CBT might rather relate to fear extinction processes and might not even be specific to anxiety disorders. Similarly, the lack of overlap between the CpGs predicting treatment response at POST and FU may reflect distinct molecular mechanisms underlying short- and long-term clinical effects of CBT. While post-treatment response might be linked to mechanisms facilitating new associative learning, cognitive restructuring and fear extinction, those associated with response at FU may rather reflect processes related to the consolidation of therapeutic gains over time. Given that lasting behavioral change involves memory-related processes, including fear extinction and the stabilization of newly learned adaptive responses, it is plausible that different epigenetic marks are relevant for immediate versus long-term treatment success.

### Limitations

Despite several strengths such as the largest sample size in a longitudinal treatment context so far, rigorous inclusion and exclusion criteria minimizing potential confounder effects, a highly standardized psychotherapeutic regime and a longitudinal follow-up over the course of six months, the present results ought to be interpreted in the light of some limitations:

In line with Hettema et al. [25], the present study applied a cross-disorder approach considering several anxiety disorders (PD, AG, SAD and SP) jointly. This approach was chosen based on evidence for an extensive clinical and biological overlap between anxiety disorders, with a shared genetic component between anxiety disorders being larger than the unique contributions to any one disorder [27–32]. Therefore, subgroup effects specific to particular clinical anxiety disorder phenotypes might have remained undetected and warrant elucidation in adequately powered follow-up studies. Furthermore, although a *post hoc* analysis adjusted for comorbidity with depression did not reveal a major impact of depression on the present case-control results, with 148 CpGs and 324 CpGs remaining significantly or suggestively associated with anxiety disorders, depression might still constitute a potential mediator or confounder [74]. However, as anxiety and depressive disorders are clinically highly comorbid, the present findings overlapping between anxiety and depression (Supplementary Table S1) might be greatly informative in the search for epigenetic biomarkers of a mixed phenotype. On another note, since no data regarding smoking behavior were available for healthy controls, the case-control analysis could not be controlled for a potential influence of smoking. However, when cross-referencing the presently identified significant hits with CpGs reported to be associated with smoking in a recent EWAS [45] and in an EWAS of smoking in the patient subgroup, no overlaps could be identified. Similarly, while substance abuse disorders including alcohol use disorder constituted an exclusion criterion, alcohol consumption in terms of drinks/day or mg alcohol/day or lifestyle factors, such as BMI, that are known to influence DNA methylation [75–80], were not controlled for. Future studies with larger and thus sufficiently powered sample sizes could investigate the epigenetic overlap between anxiety disorders and their comorbidities as well as lifestyle factors, and additionally perform sex-specific analyses, as sexually dimorphic DNA methylation has been suggested to determine sex-/gender-specific differences with respect to disease susceptibility, environmental exposure or therapeutic interventions [81, 82]. Furthermore, no randomized waitlist-controlled study design was applied, which precludes the unequivocal conclusion of the presently observed longitudinal changes in DNA methylation being due to psychotherapy effects only. Similarly, some patients were medicated with mostly antidepressants at baseline. Therefore, it cannot be disentangled whether the DNA methylation patterns presently observed to predict treatment response and the DNA methylation changes along with treatment response are mechanistically related to CBT alone or a combination of CBT and medication effects. A medication effect alone, however, is improbable as medication dosage had to remain unchanged throughout the trial [34] and the majority of patients (76%) was unmedicated. Along those lines, a confounding influence of antidepressant medication on the present case-control EWAS results cannot be fully excluded [83], although medication had to be unaltered for at least three months before study inclusion in case of a newly introduced medication and for at least two months in case of dosage adaptation of an existing medication [34]. Additionally, cross-referencing of the presently identified significant hits with the ten CpGs reported to be associated with antidepressant use in a recent EWAS [84] did not reveal any overlap. Also, as epigenetic processes are mechanistically located at the interface between genetic and environmental influences in anxiety disorders [17, 18, 85], environmental factors such as perinatal factors, childhood adversity, or more recent adverse life events might have confounded the present findings and should be considered systematically in futures studies [21]. On a technical note, epigenome-wide DNA methylation was determined in blood, i.e. peripheral tissue, as brain tissue cannot be ascertained in vivo in patients, particularly not in a longitudinal approach. Thus, the present results have to be considered as accessible surrogate markers and need to be followed up regarding their validity as proxies for central nervous system processes in silico (e.g., IMAGE-CpG [86], BECON [87], Blood Brain DNA Methylation Comparison Tool [88], AMAZE-CpG [89], which, however, do not cover all relevant brain regions of interest), experimentally in *post mortem* human brain tissue, or in a back-translational approach using animal models. Also, while the present analyses conducted in whole blood have been statistically corrected for cell-type composition, a targeted cell-type specific analysis might reveal distinct results for different cell types such as monocytes or granulocytes [25]. Finally, future studies are warranted to explore the role of epigenetic mechanisms other than DNA methylation such as histone modifications in anxiety-related phenotypes [90].

## Conclusion

In summary, the present longitudinal epigenome-wide association study revealed several differentially methylated CpGs – partly mapping to genes previously implicated in anxiety-, learning- or plasticity-related processes – that were significantly associated with anxiety disorders and suggested to be associated with response to CBT or to constitute core mechanisms of action of exposure-based interventions in anxiety disorders. Provided replication of these epigenetic signatures in independent studies and confirmation of their validity as accessible peripheral biomarkers, the present findings may eventually aid in identifying high-risk populations amenable to resilience-increasing preventive measures and in developing personalized and innovative treatment options for anxiety disorders.

## Supplementary information

Supplementary Table 1

Supplementary Table 2

Supplementary Table 3

Supplementary Table 4

Supplementary Table 5

Supplementary Figure 1

Title and legend to Supplementary Figure 1

## Data Availability

Full summary results are available upon request from the corresponding author.
