# Supplementary material for: Epigenetic markers of disease risk and psychotherapy response in anxiety disorders – a longitudinal analysis of the DNA methylome
Source: Mol Psychiatry. 2025 Apr 25;30(10):4529–42. doi: 10.1038/s41380-025-03038-5 (PMC12436192; doi:10.1038/s41380-025-03038-5)
Supplement: Supplementary file 1 — Supplementary Table 1 [file 41380_2025_3038_MOESM1_ESM.docx]

**Supplementary Table S1:** Epigenome-wide significantly differentially methylated CpG sites (p≤6.409e-08) not remaining significantly associated with anxiety disorders after statistical adjustment for comorbidity with depression in patients with anxiety disorders (N=378) vs. healthy controls (N=295).

| **CpG** | **T-Value** | **P-Value** | **Mean of Patients - Controls (Δβ)** | **Nearest Gene Annotation** |
| --- | --- | --- | --- | --- |
| cg16928523 ^a)^ | -6.98 | 7.88e-12 | -0.004 (-0.72%) | *LOC389602* |
| cg23966473 ^a)^ | -6.65 | 6.21e-11 | -0.006 (-0.83%) | *SPRN* |
| cg05812008 ^a)^ | -6.65 | 6.29e-11 | -0.016 (-3.74%) | *SNHG16* |
| cg20928554 ^a)^ | -6.62 | 7.82e-11 | -0.014 (-1.85%) | *LY9* |
| cg13629798 ^a)^ | -6.61 | 8.67e-11 | -0.001 (-0.08%) | *MAPKAPK3* |
| cg20171451 ^a)^ | -6.6 | 9.07e-11 | -0.012 (-2.75%) | *CSTL1* |
| cg03041730 ^a)^ | -6.58 | 9.52e-11 | -0.003 (-0.36%) | *GAK* |
| cg09775795 ^a)^ | -6.57 | 1.02e-10 | -0.01 (-1.41%) | *DYRK1A* |
| cg27190839 ^a)^ | -6.56 | 1.11e-10 | -0.012 (-1.65%) | *LINC00294* |
| cg19977004 ^a)^ | -6.56 | 1.12e-10 | -0.004 (-0.48%) | *BRSK2* |
| cg04764687 ^a)^ | -6.54 | 1.29e-10 | -0.005 (-0.84%) | *TCF25* |
| cg26933384 ^a)^ | -6.54 | 1.31e-10 | -0.003 (-0.43%) | *NUMBL* |
| cg04194457 ^a)^ | -6.53 | 1.36e-10 | -0.005 (-0.61%) | *SUMO1P1* |
| cg11683966 ^a)^ | -6.52 | 1.47e-10 | -0.004 (-0.54%) | *SLC25A3P1* |
| cg17928916 ^a)^ | -6.5 | 1.63e-10 | -0.004 (-0.49%) | *FASN* |
| cg06686789 ^a)^ | -6.48 | 1.83e-10 | -0.024 (-4%) | *SLC18A1* |
| cg04754212 ^a)^ | -6.46 | 2.05e-10 | -0.009 (-1.29%) | *UROS* |
| cg24978424 ^a)^ | 6.44 | 2.37e-10 | 0.001 (0.85%) | *YTHDC1* |
| cg16435426 ^a)^ | -6.42 | 2.76e-10 | -0.004 (-0.57%) | *MYO1B* |
| cg01821656 ^a)^ | -6.41 | 2.97e-10 | -0.009 (-1.29%) | *MYT1L* |
| cg21251926 ^a)^ | -6.4 | 2.97e-10 | -0.005 (-0.6%) | *GML* |
| cg17232014 ^a)^ | 6.38 | 3.47e-10 | 0.005 (2.51%) | *HEBP1* |
| cg02173453 ^a)^ | -6.36 | 3.94e-10 | -0.001 (-0.17%) | *GUCY1B3* |
| cg03100611 ^a)^ | -6.35 | 4.14e-10 | -0.004 (-0.65%) | *COL5A1* |
| cg05797914 ^a)^ | -6.35 | 4.18e-10 | -0.026 (-4.51%) | *CAMK4* |
| cg05373655 ^a)^ | -6.33 | 4.82e-10 | -0.021 (-3.47%) | *MIR4666A* |
| cg25733599 ^a)^ | -6.33 | 4.93e-10 | -0.009 (-1.6%) | *MIR205HG* |
| cg23415810 ^a)^ | -6.32 | 4.94e-10 | -0.011 (-1.39%) | *ATF7* |
| cg09412808 ^a)^ | -6.32 | 4.99e-10 | -0.009 (-1.04%) | *KIAA1211* |
| cg00634941 ^a)^ | -6.3 | 5.96e-10 | -0.013 (-1.9%) | *ABCB1* |
| cg03932842 ^a)^ | -6.28 | 6.16e-10 | -0.011 (-1.74%) | *TPST1* |
| cg13803071 ^a)^ | 6.28 | 6.30e-10 | 0 (-0.11%) | *YWHAQ* |
| cg02826687 ^a)^ | -6.27 | 6.80e-10 | -0.012 (-2.17%) | *PMP2* |
| cg07582070 ^a)^ | -6.25 | 7.68e-10 | -0.008 (-1%) | *COX7A2* |
| cg01664843 ^a)^ | -6.23 | 8.20e-10 | -0.012 (-1.55%) | *C16orf74* |
| cg09463890 ^a)^ | -6.23 | 8.52e-10 | -0.018 (-2.75%) | *TBC1D1* |
| cg10506618 ^a)^ | -6.23 | 8.68e-10 | 0 (0.02%) | *TRAK1* |
| cg08219991 ^a)^ | -6.22 | 9.07e-10 | -0.002 (-0.21%) | *CCDC85C* |
| cg07699673 ^a)^ | -6.22 | 9.13e-10 | -0.013 (-1.96%) | *TTC39B* |
| cg11236401 ^a)^ | -6.2 | 1.02e-09 | -0.011 (-1.43%) | *USP1* |
| cg01489981 ^a)^ | -6.19 | 1.09e-09 | -0.005 (-0.61%) | *TSG101* |
| cg19190632 ^a)^ | -6.18 | 1.10e-09 | -0.01 (-1.43%) | *PDLIM1* |
| cg18438461 ^a)^ | -6.18 | 1.19e-09 | -0.011 (-1.46%) | *PXDN* |
| cg14067657 ^a)^ | -6.17 | 1.22e-09 | -0.004 (-0.5%) | *PMEPA1* |
| cg06471677 ^a)^ | -6.16 | 1.33e-09 | -0.011 (-1.33%) | *RFWD2* |
| cg17419329 ^a)^ | -6.15 | 1.33e-09 | -0.014 (-2.22%) | *CAB39* |
| cg11352339 ^a)^ | -6.15 | 1.34e-09 | -0.012 (-1.53%) | *YIPF3* |
| cg12582138 ^a)^ | -6.15 | 1.35e-09 | -0.009 (-1%) | *PDK2* |
| cg23409591 ^a)^ | -6.15 | 1.38e-09 | -0.005 (-0.62%) | *TNP1* |
| cg16202803 ^a)^ | -6.15 | 1.38e-09 | -0.002 (-0.21%) | *HDAC4* |
| cg22754654 ^a)^ | -6.14 | 1.47e-09 | -0.008 (-1.16%) | *SAMD1* |
| cg06957130 ^a)^ | 6.13 | 1.58e-09 | 0.004 (3.72%) | *CELSR1* |
| cg19317565 ^a)^ | -6.12 | 1.65e-09 | -0.015 (-2.39%) | *TCF7L1* |
| cg27625055 ^a)^ | 6.11 | 1.70e-09 | 0.005 (7.22%) | *PTPRT* |
| cg22375623 ^a)^ | -6.09 | 1.95e-09 | -0.011 (-1.31%) | *IDH3A* |
| cg06980445 ^a)^ | -6.08 | 2.05e-09 | 0.003 (0.43%) | *ZNF691* |
| cg27052090 ^a)^ | -6.08 | 2.05e-09 | -0.007 (-0.9%) | *GMFB* |
| cg05445097 ^a)^ | -6.08 | 2.09e-09 | -0.019 (-2.39%) | *DLG5* |
| cg08968899 ^a)^ | -6.08 | 2.09e-09 | -0.013 (-1.64%) | *MATN2* |
| cg26296524 ^a)^ | -6.07 | 2.23e-09 | -0.011 (-1.68%) | *KAZN* |
| cg12985204 ^a)^ | -6.07 | 2.24e-09 | -0.008 (-1.01%) | *DSCAML1* |
| cg26107100 ^a)^ | -6.07 | 2.26e-09 | -0.002 (-0.24%) | *CCDC88B* |
| cg11644394 ^a)^ | -6.06 | 2.28e-09 | -0.026 (-4.47%) | *TPK1* |
| cg04955246 ^a)^ | -6.07 | 2.34e-09 | -0.002 (-0.34%) | *PRKCA* |
| cg01826858 ^a)^ | -6.06 | 2.41e-09 | -0.004 (-0.67%) | *FBXL19* |
| cg22361604 ^a)^ | -6.05 | 2.41e-09 | -0.017 (-2.67%) | *NFIA-AS2* |
| cg22351824 ^a)^ | -6.05 | 2.45e-09 | -0.01 (-1.85%) | *ZNF70* |
| cg00423987 ^a)^ | -6.04 | 2.52e-09 | -0.012 (-1.53%) | *MSH2* |
| cg12059309 ^a)^ | -6.05 | 2.56e-09 | -0.001 (-0.12%) | *CNOT2* |
| cg16602927 ^a)^ | -6.04 | 2.63e-09 | -0.01 (-1.28%) | *NUP54* |
| cg24920997 ^a)^ | -6.04 | 2.65e-09 | -0.019 (-3.47%) | *FGF12* |
| cg15052719 ^a)^ | -6.04 | 2.69e-09 | -0.017 (-2.51%) | *VIPR2* |
| cg02084259 ^a)^ | -6.03 | 2.73e-09 | -0.007 (-0.9%) | *AVEN* |
| cg12042864 ^a)^ | -6.04 | 2.74e-09 | -0.005 (-0.65%) | *ATP5J2-PTCD1* |
| cg17906245 ^a)^ | -6.02 | 2.93e-09 | -0.012 (-1.57%) | *SPATA45* |
| cg13217994 ^a)^ | -6.01 | 3.07e-09 | -0.006 (-0.69%) | *CTDP1* |
| cg24517611 ^a)^ | -6.01 | 3.11e-09 | -0.008 (-0.93%) | *NUP85* |
| cg09042733 ^a)^ | -6.01 | 3.13e-09 | -0.006 (-0.66%) | *PLEKHB2* |
| cg14290775 ^a)^ | -6.01 | 3.17e-09 | -0.021 (-3.5%) | *SLC39A11* |
| cg07053726 ^a)^ | -6.01 | 3.17e-09 | -0.001 (-0.12%) | *PCNX1* |
| cg24069126 ^a)^ | -6.01 | 3.22e-09 | -0.009 (-1.18%) | *PTPN1* |
| cg09217023 ^a)^ | -006 | 3.32e-09 | -0.012 (-1.99%) | *THUMPD3* |
| cg24679077 ^a)^ | -006 | 3.40e-09 | -0.001 (-0.14%) | *ZSCAN16-AS1* |
| cg22839188 ^a)^ | -5.99 | 3.53e-09 | -0.013 (-1.76%) | *TLE1* |
| cg06073004 ^a)^ | -5.99 | 3.58e-09 | -0.005 (-0.67%) | *FHAD1* |
| cg13312309 ^a)^ | -5.98 | 3.61e-09 | -0.01 (-1.22%) | *PLCG1* |
| cg13788717 ^a)^ | -5.98 | 3.63e-09 | -0.003 (-0.37%) | *TULP2* |
| cg24597989 ^b)^ | -5.98 | 3.74e-09 | -0.001 (-0.08%) | *PLLP* |
| cg16374348 ^a)^ | -5.98 | 3.78e-09 | -0.006 (-0.83%) | *MTIF3* |
| cg13707777 ^a)^ | -5.98 | 3.81e-09 | -0.009 (-1.13%) | *PDCD6* |
| cg23187669 ^a)^ | -5.98 | 3.84e-09 | -0.011 (-1.53%) | *MFAP3L* |
| cg01211165 ^a)^ | -5.97 | 3.97e-09 | -0.012 (-1.47%) | *ST18* |
| cg21406869 ^a)^ | -5.97 | 3.98e-09 | -0.012 (-1.41%) | *PIAS1* |
| cg19774484 ^a)^ | -5.98 | 4.01e-09 | -0.004 (-0.49%) | *RUNX1* |
| cg12900510 ^a)^ | 5.96 | 4.12e-09 | 0.002 (4.04%) | *KIAA0895L* |
| cg01379428 ^a)^ | -5.96 | 4.14e-09 | -0.01 (-1.44%) | *TRIM52-AS1* |
| cg17947121 ^a)^ | -5.96 | 4.21e-09 | -0.008 (-1%) | *KPRP* |
| cg10219156 ^a)^ | -5.96 | 4.33e-09 | -0.004 (-0.49%) | *SSUH2* |
| cg00836302 ^a)^ | -5.96 | 4.37e-09 | -0.013 (-2.18%) | *ADAMTSL5* |
| cg22980611 ^a)^ | 5.95 | 4.38e-09 | 0.004 (6.76%) | *DNAJB2* |
| cg15817495 ^a)^ | -5.95 | 4.45e-09 | -0.001 (-0.15%) | *MAN1A2* |
| cg26642667 ^a)^ | 5.95 | 4.48e-09 | 0.01 (5.86%) | *SND1* |
| cg18418833 ^a)^ | -5.94 | 4.53e-09 | -0.006 (-0.75%) | *USP43* |
| cg10350880 ^a)^ | -5.95 | 4.62e-09 | -0.012 (-1.61%) | *EPHB4* |
| cg00500027 ^a)^ | -5.94 | 4.65e-09 | -0.01 (-1.28%) | *OTOF* |
| cg20731100 ^a)^ | -5.94 | 4.81e-09 | -0.013 (-1.74%) | *TNS3* |
| cg05704926 ^a)^ | -5.93 | 4.85e-09 | -0.005 (-0.68%) | *ARL4C* |
| cg04112800 ^a)^ | -5.93 | 4.95e-09 | 0 (0.04%) | *ZNF341* |
| cg02986878 ^a)^ | -5.93 | 5.07e-09 | -0.013 (-1.74%) | *WHRN* |
| cg14817448 ^a)^ | -5.93 | 5.10e-09 | -0.013 (-2.38%) | *ZNF148* |
| cg01624068 ^a)^ | -5.93 | 5.12e-09 | -0.009 (-2.03%) | *TUBGCP3* |
| cg15097551 ^b)^ | -5.92 | 5.38e-09 | -0.001 (-0.14%) | *RFC2* |
| cg26507853 ^a)^ | -5.91 | 5.44e-09 | -0.007 (-0.88%) | *SRD5A3* |
| cg14541281 ^a)^ | -5.92 | 5.65e-09 | -0.013 (-1.58%) | *POLR3E* |
| cg20228495 ^a)^ | -5.91 | 5.69e-09 | 0 (-0.02%) | *SEMA4F* |
| cg04084600 ^a)^ | -5.91 | 5.71e-09 | -0.011 (-1.97%) | *SPP2* |
| cg12126519 ^a)^ | -5.9 | 5.76e-09 | -0.004 (-0.77%) | *LPP* |
| cg09448460 ^a)^ | -5.91 | 5.79e-09 | -0.011 (-3.19%) | *GPR1-AS* |
| cg01365261 ^a)^ | -5.9 | 5.84e-09 | -0.009 (-1.15%) | *ZMYM1* |
| cg11824509 ^a)^ | -5.9 | 5.91e-09 | -0.01 (-1.33%) | *TMEM254-AS1* |
| cg18893857 ^a)^ | 5.91 | 5.99e-09 | 0.005 (8.46%) | *MIR193BHG* |
| cg21720385 ^a)^ | -5.9 | 6.15e-09 | -0.003 (-0.32%) | *UBXN6* |
| cg01958008 ^a)^ | -5.9 | 6.16e-09 | -0.016 (-2.21%) | *ACAD9* |
| cg18343870 ^a)^ | -5.89 | 6.17e-09 | -0.009 (-1.16%) | *PLXNC1* |
| cg10194352 ^a)^ | -5.89 | 6.17e-09 | -0.013 (-2.23%) | *MMP2-AS1* |
| cg01679663 ^a)^ | -5.89 | 6.27e-09 | -0.005 (-0.77%) | *BCKDHB* |
| cg09124518 ^a)^ | -5.89 | 6.39e-09 | -0.002 (-0.24%) | *STK32C* |
| cg16695999 ^a)^ | -5.89 | 6.41e-09 | -0.003 (-0.36%) | *KIAA0825* |
| cg15864790 ^a)^ | -5.89 | 6.44e-09 | -0.01 (-1.24%) | *SLC35F5* |
| cg22355691 ^a)^ | -5.88 | 6.50e-09 | -0.012 (-1.71%) | *ZNF791* |
| cg05035616 ^a)^ | -5.89 | 6.50e-09 | -0.002 (-0.33%) | *GNAO1* |
| cg02052377 ^a)^ | -5.88 | 6.52e-09 | -0.014 (-1.98%) | *MRVI1* |
| cg27374674 ^a)^ | -5.88 | 6.59e-09 | 0.001 (0.1%) | *GAS7* |
| cg06515543 ^a)^ | -5.88 | 6.66e-09 | -0.004 (-0.58%) | *NRTN* |
| cg11960355 ^a)^ | -5.87 | 6.82e-09 | -0.015 (-2.4%) | *LINC00114* |
| cg06840167 ^a)^ | -5.88 | 6.83e-09 | -0.01 (-1.21%) | *FAM151B* |
| cg12816829 ^a)^ | -5.87 | 6.88e-09 | -0.009 (-1.16%) | *TRH* |
| cg21849780 ^a)^ | -5.87 | 7.02e-09 | -0.004 (-0.53%) | *UNC50* |
| cg26743529 ^a)^ | -5.87 | 7.10e-09 | -0.017 (-2.77%) | *C10orf126* |
| cg13315166 ^a)^ | -5.87 | 7.28e-09 | 0.002 (0.23%) | *MYO18A* |
| cg24851906 ^a)^ | -5.86 | 7.36e-09 | -0.004 (-0.56%) | *LOC105378137* |
| cg11265952 ^a)^ | -5.86 | 7.41e-09 | -0.013 (-1.63%) | *HESX1* |
| cg19268708 ^a)^ | -5.86 | 7.47e-09 | -0.005 (-0.68%) | *ACTRT2* |
| cg20672245 ^a)^ | -5.86 | 7.47e-09 | -0.002 (-0.31%) | *ATP5J2-PTCD1* |
| cg03808418 ^a)^ | -5.86 | 7.64e-09 | -0.012 (-2.01%) | *KCNV2* |
| cg06895230 ^a)^ | -5.85 | 7.65e-09 | -0.004 (-0.59%) | *PVT1* |
| cg12798675 ^a)^ | -5.86 | 7.70e-09 | -0.019 (-3.04%) | *LINC00239* |
| cg07844072 ^a)^ | -5.85 | 7.91e-09 | -0.01 (-1.24%) | *MUM1* |
| cg12026546 ^a)^ | -5.85 | 8.02e-09 | -0.002 (-0.26%) | *INPP5A* |
| cg24627737 ^a)^ | -5.84 | 8.09e-09 | -0.015 (-2.03%) | *MSMO1* |
| cg09901100 ^a)^ | -5.84 | 8.14e-09 | -0.005 (-0.55%) | *WDR91* |
| cg20559736 ^a)^ | -5.84 | 8.28e-09 | -0.018 (-2.64%) | *CEACAM19* |
| cg24524245 ^a)^ | -5.84 | 8.47e-09 | -0.01 (-1.71%) | *CLDN23* |
| cg10279667 ^a)^ | -5.84 | 8.49e-09 | -0.013 (-1.92%) | *KNTC1* |
| cg03317280 ^a)^ | 5.84 | 8.57e-09 | 0.004 (5.12%) | *RNF187* |
| cg03395088 ^a)^ | -5.83 | 8.62e-09 | -0.003 (-0.32%) | *HDAC4* |
| cg26664492 ^a)^ | -5.84 | 8.72e-09 | -0.004 (-0.46%) | *LINC00964* |
| cg21298408 ^a)^ | -5.83 | 8.81e-09 | -0.005 (-0.72%) | *NAT1* |
| cg04415694 ^a)^ | -5.82 | 9.37e-09 | -0.001 (-0.11%) | *CTNNB1* |
| cg12724894 ^a)^ | -5.82 | 9.52e-09 | -0.014 (-2.71%) | *DIP2C* |
| cg06616055 ^a)^ | -5.82 | 9.67e-09 | -0.009 (-1.23%) | *CDHR5* |
| cg08558652 ^a)^ | -5.81 | 9.70e-09 | -0.008 (-1.03%) | *LONP2* |
| cg14470485 ^a)^ | -5.81 | 9.70e-09 | -0.003 (-0.43%) | *GUCA1B* |
| cg07680195 ^a)^ | 5.81 | 9.75e-09 | 0.001 (0.25%) | *PGLS* |
| cg13580380 ^a)^ | -5.81 | 9.84e-09 | -0.004 (-0.54%) | *MSC* |
| cg22779765 ^a)^ | -5.81 | 9.88e-09 | -0.003 (-0.37%) | *TRAF3* |
| cg17004038 ^a)^ | -5.81 | 9.91e-09 | -0.007 (-0.8%) | *ALKBH2* |
| cg08355228 ^a)^ | -5.81 | 1.00e-08 | -0.009 (-1.57%) | *LOC101927847* |
| cg07023901 ^a)^ | -5.81 | 1.01e-08 | -0.015 (-2.18%) | *LINC02245* |
| cg03027547 ^a)^ | -5.8 | 1.02e-08 | -0.005 (-0.72%) | *PLEKHG1* |
| cg04782472 ^a)^ | -5.8 | 1.05e-08 | -0.004 (-0.52%) | *C7orf50* |
| cg24736345 ^a)^ | -5.8 | 1.08e-08 | -0.001 (-0.08%) | *GNA11* |
| cg00291316 ^a)^ | -5.8 | 1.08e-08 | -0.006 (-0.72%) | *RP1L1* |
| cg15914307 ^a)^ | -5.79 | 1.10e-08 | -0.009 (-1.95%) | *ARRDC3-AS1* |
| cg22010309 ^a)^ | -5.79 | 1.10e-08 | -0.002 (-0.25%) | *MIR1269A* |
| cg14020598 ^a)^ | -5.79 | 1.11e-08 | -0.011 (-1.79%) | *LINCMD1* |
| cg24384817 ^a)^ | -5.79 | 1.12e-08 | -0.005 (-0.65%) | *FCHSD1* |
| cg25151926 ^a)^ | -5.79 | 1.14e-08 | -0.011 (-1.86%) | *LINC02110* |
| cg05097579 ^a)^ | -5.78 | 1.14e-08 | -0.008 (-1.16%) | *IL20RB* |
| cg19434769 ^a)^ | -5.78 | 1.16e-08 | -0.015 (-2.23%) | *NHSL1* |
| cg12753804 ^a)^ | -5.78 | 1.16e-08 | -0.006 (-0.72%) | *LPP* |
| cg02171258 ^b)^ | -5.78 | 1.17e-08 | -0.006 (-0.78%) | *NT5DC1* |
| cg26444282 ^a)^ | -5.78 | 1.19e-08 | -0.004 (-0.47%) | *SYNPO* |
| cg00730348 ^a)^ | -5.77 | 1.21e-08 | -0.012 (-1.89%) | *RNASEH2B-AS1* |
| cg17370785 ^a)^ | -5.77 | 1.25e-08 | -0.002 (-0.31%) | *MXD4* |
| cg13335769 ^a)^ | -5.77 | 1.25e-08 | -0.007 (-0.98%) | *VPS52* |
| cg19788417 ^a)^ | -5.77 | 1.26e-08 | -0.013 (-2.36%) | *DEFB123* |
| cg12629012 ^a)^ | -5.77 | 1.29e-08 | -0.006 (-0.73%) | *REST* |
| cg23587044 ^a)^ | -5.77 | 1.30e-08 | -0.017 (-3.61%) | *DLG1* |
| cg22695245 ^a)^ | -5.76 | 1.35e-08 | -0.005 (-0.58%) | *TRDMT1* |
| cg17858098 ^a)^ | -5.75 | 1.39e-08 | -0.012 (-1.6%) | *ECH1* |
| cg16607181 ^a)^ | -5.75 | 1.42e-08 | -0.004 (-0.41%) | *LOC105378146* |
| cg19188370 ^a)^ | -5.75 | 1.42e-08 | -0.012 (-1.45%) | *NCOR2* |
| cg05714008 ^a)^ | -5.74 | 1.44e-08 | 0.005 (0.89%) | *GRHL2* |
| cg02201969 ^a)^ | -5.74 | 1.47e-08 | -0.002 (-0.19%) | *TBC1D14* |
| cg02895802 ^a)^ | -5.74 | 1.52e-08 | -0.006 (-0.76%) | *OLA1* |
| cg02330195 ^a)^ | -5.73 | 1.52e-08 | -0.007 (-1.12%) | *CDH23* |
| cg13278115 ^a)^ | -5.73 | 1.55e-08 | -0.001 (-0.12%) | *KCNK12* |
| cg08136599 ^a)^ | -5.73 | 1.57e-08 | -0.008 (-1.03%) | *BLOC1S5-TXNDC5* |
| cg26609894 ^a)^ | -5.73 | 1.57e-08 | -0.008 (-0.97%) | *CERS4* |
| cg24619785 ^a)^ | -5.73 | 1.57e-08 | -0.004 (-0.47%) | *ASAP2* |
| cg22024692 ^a)^ | -5.72 | 1.61e-08 | -0.001 (-0.1%) | *B3GNTL1* |
| cg13339291 ^a)^ | -5.72 | 1.62e-08 | -0.008 (-1.06%) | *CCDC57* |
| cg11992263 ^a)^ | -5.72 | 1.63e-08 | -0.006 (-1.11%) | *MIR4454* |
| cg04488145 ^a)^ | -5.72 | 1.63e-08 | -0.003 (-0.31%) | *MYL3* |
| cg16205325 ^a)^ | 5.72 | 1.65e-08 | 0.004 (4.04%) | *MAP4* |
| cg01441738 ^a)^ | -5.72 | 1.66e-08 | -0.007 (-0.99%) | *ACBD6* |
| cg20573956 ^a)^ | -5.72 | 1.67e-08 | -0.014 (-2.37%) | *BOC* |
| cg27151629 ^a)^ | 5.72 | 1.70e-08 | 0.008 (6.55%) | *SLC47A1* |
| cg24366105 ^a)^ | -5.72 | 1.71e-08 | -0.003 (-0.44%) | *SNX8* |
| cg20455436 ^a)^ | -5.71 | 1.73e-08 | -0.003 (-0.36%) | *ZNF628* |
| cg03330642 ^a)^ | -5.71 | 1.74e-08 | -0.008 (-1.54%) | *GRM6* |
| cg12290615 ^a)^ | -5.71 | 1.75e-08 | -0.027 (-5.4%) | *SMAP1* |
| cg00303641 ^a)^ | -5.71 | 1.76e-08 | -0.013 (-1.79%) | *ERN2* |
| cg15789385 ^a)^ | -5.7 | 1.80e-08 | -0.013 (-1.92%) | *KDM2B* |
| cg05348421 ^a)^ | 5.7 | 1.84e-08 | 0.01 (3.4%) | *CRK* |
| cg22905192 ^a)^ | -5.7 | 1.84e-08 | -0.01 (-1.53%) | *GSX2* |
| cg08739385 ^a)^ | -5.7 | 1.85e-08 | -0.014 (-2.04%) | *EPHA10* |
| cg10197405 ^a)^ | -5.7 | 1.85e-08 | -0.011 (-1.99%) | *PRSS8* |
| cg16862869 ^b)^ | -5.7 | 1.86e-08 | -0.012 (-2.29%) | *DYM* |
| cg25143247 ^a)^ | -5.69 | 1.91e-08 | -0.005 (-0.64%) | *PACRG* |
| cg12164564 ^a)^ | -5.69 | 1.93e-08 | -0.005 (-0.75%) | *LRBA* |
| cg17825156 ^a)^ | 5.69 | 1.94e-08 | 0.012 (5.08%) | *MIER1* |
| cg03464143 ^a)^ | -5.69 | 1.99e-08 | -0.009 (-1.19%) | *ZNF493* |
| cg07593903 ^a)^ | -5.68 | 2.02e-08 | -0.003 (-0.42%) | *KANK4* |
| cg02240671 ^a)^ | -5.68 | 2.06e-08 | -0.014 (-2.19%) | *POC1A* |
| cg22341362 ^a)^ | -5.68 | 2.08e-08 | -0.013 (-1.82%) | *LOC101927588* |
| cg15334836 ^a)^ | -5.68 | 2.09e-08 | 0 (-0.05%) | *MELTF* |
| cg22511564 ^a)^ | -5.68 | 2.09e-08 | -0.007 (-0.92%) | *MYLK3* |
| cg14548038 ^a)^ | -5.66 | 2.25e-08 | -0.013 (-1.54%) | *TOR4A* |
| cg02600258 ^a)^ | -5.67 | 2.26e-08 | -0.015 (-2.22%) | *POC5* |
| cg11965754 ^a)^ | -5.66 | 2.26e-08 | -0.004 (-0.57%) | *KCTD14* |
| cg07851575 ^a)^ | -5.66 | 2.28e-08 | 0.001 (0.12%) | *TPTE2P5* |
| cg10655371 ^a)^ | -5.66 | 2.29e-08 | -0.008 (-1.36%) | *CYP51A1* |
| cg17698037 ^a)^ | -5.66 | 2.36e-08 | -0.005 (-0.71%) | *LPP* |
| cg03733229 ^b)^ | 5.65 | 2.40e-08 | 0.006 (5.63%) | *COL18A1* |
| cg16207974 ^a)^ | -5.65 | 2.41e-08 | -0.001 (-0.12%) | *PRELID3A* |
| cg24520968 ^a)^ | -5.65 | 2.43e-08 | -0.004 (-0.44%) | *CEP85L* |
| cg05933189 ^a)^ | -5.65 | 2.44e-08 | -0.007 (-1%) | *CSNK1G1* |
| cg25515854 ^a)^ | -5.65 | 2.47e-08 | 0.001 (0.17%) | *PEX7* |
| cg01398168 ^a)^ | -5.65 | 2.51e-08 | -0.014 (-1.99%) | *C19orf18* |
| cg01897092 ^a)^ | -5.64 | 2.51e-08 | -0.013 (-1.88%) | *RASL10B* |
| cg01032946 ^a)^ | -5.64 | 2.52e-08 | -0.002 (-0.24%) | *SETD1A* |
| cg20102393 ^a)^ | -5.64 | 2.53e-08 | -0.006 (-0.8%) | *OVGP1* |
| cg24693061 ^b)^ | -5.64 | 2.53e-08 | -0.013 (-6.95%) | *NKX6-2* |
| cg15604993 ^a)^ | -5.64 | 2.57e-08 | -0.012 (-2.02%) | *MYO5C* |
| cg04076264 ^a)^ | -5.64 | 2.57e-08 | -0.003 (-0.29%) | *PPP1R14C* |
| cg01588444 ^a)^ | -5.64 | 2.60e-08 | -0.003 (-0.33%) | *LOC101928416* |
| cg02905251 ^a)^ | -5.64 | 2.61e-08 | -0.014 (-2.09%) | *ASB18* |
| cg20259981 ^a)^ | -5.63 | 2.68e-08 | -0.001 (-0.13%) | *ST18* |
| cg14832223 ^a)^ | -5.63 | 2.68e-08 | -0.009 (-1.28%) | *C15orf39* |
| cg05585551 ^a)^ | -5.63 | 2.72e-08 | -0.004 (-0.55%) | *RPS6KA2-AS1* |
| cg11380323 ^b)^ | -5.63 | 2.74e-08 | -0.008 (-0.96%) | *RAB3GAP2* |
| cg14279539 ^a)^ | -5.63 | 2.75e-08 | -0.012 (-1.84%) | *NFATC2* |
| cg07172676 ^a)^ | -5.63 | 2.76e-08 | -0.001 (-0.17%) | *PLEC* |
| cg14161477 ^a)^ | -5.62 | 2.78e-08 | -0.001 (-0.14%) | *TMCO3* |
| cg25627341 ^a)^ | -5.62 | 2.81e-08 | 0.001 (0.19%) | *LOC100128993* |
| cg26498782 ^a)^ | -5.63 | 2.83e-08 | -0.006 (-0.85%) | *LOC102723831* |
| cg24858738 ^a)^ | 5.62 | 2.83e-08 | 0.003 (3.02%) | *RANBP9* |
| cg22732549 ^a)^ | -5.62 | 2.86e-08 | -0.014 (-1.88%) | *ERI1* |
| cg17372451 ^a)^ | -5.62 | 2.87e-08 | -0.017 (-2.3%) | *SLC15A5* |
| cg09960003 ^a)^ | -5.62 | 2.91e-08 | -0.006 (-1.02%) | *CRTAC1* |
| cg21776419 ^a)^ | -5.61 | 2.94e-08 | -0.007 (-1.06%) | *SSBP2* |
| cg03686090 ^a)^ | -5.62 | 2.95e-08 | -0.007 (-1.01%) | *ADCY6* |
| cg17921886 ^a)^ | -5.61 | 3.03e-08 | -0.009 (-1.21%) | *NR2E3* |
| cg21186779 ^a)^ | -5.61 | 3.11e-08 | -0.004 (-0.74%) | *ANKRD26P1* |
| cg10889196 ^a)^ | -5.6 | 3.12e-08 | 0 (-0.06%) | *LINC00111* |
| cg09841361 ^a)^ | -5.6 | 3.12e-08 | -0.011 (-1.49%) | *TXNRD3NB* |
| cg14590262 ^a)^ | -5.61 | 3.12e-08 | -0.006 (-0.78%) | *ZMYND8* |
| cg23132859 ^a)^ | -5.6 | 3.18e-08 | -0.007 (-1.41%) | *VPS53* |
| cg18345806 ^b)^ | 5.6 | 3.25e-08 | 0.003 (4.41%) | *NMU* |
| cg21877541 ^a)^ | -5.6 | 3.29e-08 | -0.011 (-1.62%) | *LOC100505530* |
| cg25510018 ^a)^ | -5.59 | 3.32e-08 | -0.003 (-0.31%) | *TRIP10* |
| cg04510155 ^a)^ | -5.59 | 3.40e-08 | -0.004 (-0.44%) | *TSEN54* |
| cg09431264 ^a)^ | -5.59 | 3.41e-08 | -0.003 (-0.45%) | *TJP3* |
| cg18499294 ^b)^ | -5.59 | 3.43e-08 | -0.002 (-0.38%) | *SAMD4A* |
| cg25621735 ^a)^ | -5.59 | 3.44e-08 | -0.014 (-1.91%) | *SLC16A14* |
| cg27551169 ^a)^ | -5.59 | 3.46e-08 | -0.003 (-0.29%) | *ALPK3* |
| cg01866220 ^a)^ | -5.59 | 3.47e-08 | -0.013 (-1.65%) | *NFATC2IP* |
| cg24377604 ^a)^ | -5.59 | 3.48e-08 | -0.003 (-0.4%) | *KIAA1211* |
| cg13675624 ^a)^ | -5.58 | 3.59e-08 | -0.01 (-1.33%) | *PLEKHG6* |
| cg02708904 ^a)^ | -5.58 | 3.61e-08 | -0.007 (-1.17%) | *LOC102723838* |
| cg05804856 ^a)^ | -5.58 | 3.62e-08 | -0.015 (-2.31%) | *ADAM12* |
| cg17655527 ^a)^ | -5.58 | 3.63e-08 | -0.005 (-0.6%) | *ZNF865* |
| cg07195508 ^a)^ | 5.58 | 3.66e-08 | 0.002 (3.1%) | *MIER1* |
| cg23971362 ^a)^ | -5.58 | 3.68e-08 | -0.011 (-1.68%) | *LRRC43* |
| cg05855199 ^a)^ | -5.58 | 3.70e-08 | -0.009 (-1.3%) | *CHURC1* |
| cg12573727 ^a)^ | -5.57 | 3.72e-08 | -0.004 (-0.53%) | *PALM* |
| cg09208919 ^a)^ | -5.57 | 3.74e-08 | -0.01 (-1.37%) | *DOCK8* |
| cg14669913 ^a)^ | -5.57 | 3.74e-08 | -0.006 (-0.72%) | *OSGIN2* |
| cg18889307 ^a)^ | -5.57 | 3.77e-08 | 0 (-0.01%) | *TGIF1* |
| cg13590711 ^a)^ | -5.57 | 3.81e-08 | -0.005 (-0.87%) | *PRKD1* |
| cg16827359 ^a)^ | -5.57 | 3.85e-08 | -0.011 (-1.31%) | *C22orf34* |
| cg13676431 ^a)^ | -5.56 | 3.94e-08 | -0.001 (-0.18%) | *CDCP2* |
| cg08435603 ^b)^ | -5.55 | 4.10e-08 | 0 (0.04%) | *ZNF100* |
| cg02523617 ^a)^ | 5.56 | 4.10e-08 | 0.001 (0.52%) | *UBE2K* |
| cg24912310 ^a)^ | -5.55 | 4.12e-08 | -0.006 (-0.73%) | *WDR88* |
| cg13319501 ^a)^ | -5.55 | 4.15e-08 | -0.007 (-0.88%) | *SFMBT2* |
| cg06775669 ^a)^ | -5.55 | 4.28e-08 | -0.011 (-1.3%) | *CUX1* |
| cg06922305 ^b)^ | -5.55 | 4.29e-08 | 0 (-0.06%) | *USP39* |
| cg14455590 ^a)^ | -5.55 | 4.29e-08 | -0.002 (-0.3%) | *C22orf34* |
| cg00402311 ^a)^ | -5.55 | 4.33e-08 | -0.018 (-2.91%) | *CLVS1* |
| cg11241151 ^b)^ | -5.55 | 4.38e-08 | -0.002 (-0.28%) | *KCNF1* |
| cg15197458 ^a)^ | -5.54 | 4.43e-08 | -0.005 (-0.56%) | *NFIC* |
| cg15132055 ^a)^ | -5.53 | 4.67e-08 | -0.006 (-0.77%) | *APOM* |
| cg09834444 ^a)^ | -5.53 | 4.67e-08 | -0.01 (-1.49%) | *SPIRE2* |
| cg02120513 ^a)^ | -5.53 | 4.74e-08 | -0.002 (-0.26%) | *SLC1A3* |
| cg19519630 ^a)^ | -5.53 | 4.80e-08 | -0.003 (-0.39%) | *TP73* |
| cg18011945 ^a)^ | -5.53 | 4.82e-08 | -0.006 (-0.91%) | *SNRPE* |
| cg26880777 ^a)^ | -5.52 | 4.83e-08 | -0.011 (-1.65%) | *MUL1* |
| cg26494252 ^a)^ | -5.52 | 4.91e-08 | -0.003 (-0.48%) | *SLC1A6* |
| cg17307811 ^a)^ | -5.52 | 5.01e-08 | -0.016 (-2.35%) | *UBE2S* |
| cg14056065 ^b)^ | -5.52 | 5.07e-08 | -0.007 (-0.86%) | *NCR3* |
| cg06944096 ^a)^ | -5.51 | 5.13e-08 | -0.009 (-1.1%) | *RFLNA* |
| cg16767327 ^a)^ | -5.51 | 5.17e-08 | -0.012 (-1.64%) | *CLIP4* |
| cg06114440 ^a)^ | -5.51 | 5.18e-08 | 0 (-0.03%) | *PAEP* |
| cg18792381 ^a)^ | -5.51 | 5.21e-08 | -0.012 (-4.54%) | *GFPT2* |
| cg25719374 ^a)^ | -5.51 | 5.24e-08 | -0.016 (-2.66%) | *MIR1268A* |
| cg17611512 ^a)^ | -5.51 | 5.24e-08 | -0.004 (-0.42%) | *COL18A1* |
| cg05225096 ^a)^ | -5.51 | 5.25e-08 | -0.008 (-1.03%) | *C6orf106* |
| cg11224062 ^a)^ | -5.51 | 5.27e-08 | -0.002 (-0.22%) | *CCDC163* |
| cg12782201 ^a)^ | -5.51 | 5.30e-08 | -0.003 (-0.35%) | *MIR412* |
| cg23330385 ^a)^ | -5.51 | 5.31e-08 | -0.013 (-1.58%) | *ZC3H15* |
| cg13475977 ^b)^ | -5.51 | 5.38e-08 | -0.003 (-0.29%) | *CRYL1* |
| cg13977779 ^a)^ | -5.5 | 5.42e-08 | -0.002 (-0.3%) | *LOC401557* |
| cg03849780 ^a)^ | -5.51 | 5.45e-08 | 0.002 (0.31%) | *CAMTA1* |
| cg23179326 ^b)^ | -5.5 | 5.48e-08 | -0.004 (-0.58%) | *ZDHHC1* |
| cg15849237 ^b)^ | -5.5 | 5.51e-08 | -0.006 (-0.74%) | *PREX1* |
| cg06442378 ^a)^ | -5.5 | 5.52e-08 | -0.01 (-1.56%) | *PARD3* |
| cg12221713 ^a)^ | -5.49 | 5.68e-08 | -0.004 (-0.57%) | *ZFYVE28* |
| cg14212045 ^a)^ | -5.5 | 5.70e-08 | -0.012 (-1.61%) | *ARHGAP26* |
| cg25472172 ^a)^ | -5.49 | 5.72e-08 | -0.007 (-0.98%) | *GREB1* |
| cg23848181 ^a)^ | -5.49 | 5.79e-08 | -0.005 (-0.64%) | *NLRC5* |
| cg22675207 ^a)^ | -5.49 | 5.79e-08 | -0.003 (-0.31%) | *TRIM62* |
| cg09348353 ^b)^ | -5.49 | 5.79e-08 | 0 (0.03%) | *MALT1* |
| cg21147282 ^b)^ | -5.49 | 5.91e-08 | -0.004 (-0.54%) | *HDAC9* |
| cg08270825 ^a)^ | -5.49 | 5.93e-08 | -0.014 (-2.85%) | *OR1K1* |
| cg01794812 ^a)^ | -5.48 | 5.98e-08 | -0.007 (-1.1%) | *ZNF517* |
| cg23499846 ^b)^ | -5.49 | 6.00e-08 | -0.005 (-0.93%) | *CLUH* |
| cg16284395 ^a)^ | -5.48 | 6.07e-08 | -0.005 (-0.6%) | *ASB2* |
| cg15547703 ^b)^ | -5.48 | 6.21e-08 | -0.003 (-0.34%) | *CSTB* |
| cg14165528 ^a)^ | -5.48 | 6.24e-08 | -0.014 (-1.89%) | *LINC01698* |
| cg26905303 ^a)^ | -5.48 | 6.26e-08 | 0 (0.05%) | *VPS8* |
| cg27658503 ^a)^ | -5.48 | 6.29e-08 | -0.012 (-1.85%) | *LINC01448* |
| cg07925549 ^b)^ | -5.48 | 6.30e-08 | -0.01 (-1.34%) | *KRT75* |
| cg02951457 ^a)^ | -5.48 | 6.32e-08 | -0.015 (-2.35%) | *PPP1R14D* |
| cg26616537 ^b)^ | -5.47 | 6.37e-08 | -0.018 (-2.91%) | *MRAP* |

Legend to Supplementary Tab. S1: ^a)^ CpGs remaining to be suggestively associated with anxiety disorders after statistical adjustment for comorbidity with depression (defined as diagnosis of major depressive disorder, single episode or recurrent); ^b)^ CpGs not associated with anxiety disorders any more after statistical adjustment for comorbidity with depression; Δβ: Positive values indicate methylation in anxiety disorder patients > methylation in healthy controls, negative values indicate methylation in anxiety disorder patients < methylation in healthy controls.
