# Supplementary material for: Epigenetic markers of disease risk and psychotherapy response in anxiety disorders – a longitudinal analysis of the DNA methylome
Source: Mol Psychiatry. 2025 Apr 25;30(10):4529–42. doi: 10.1038/s41380-025-03038-5 (PMC12436192; doi:10.1038/s41380-025-03038-5)
Supplement: Supplementary file 2 — Supplementary Table 2 [file 41380_2025_3038_MOESM2_ESM.docx]

**Supplementary Table S2:** Epigenome-wide significantly differentially methylated CpG sites (p_Bonferroni_<0.05) associated with smoking status in patients with anxiety disorders (N=378).

| **CpG** | **Effect** | **Standard Error** | **P-Value** | **N** | **Mean Methylation** | **Bonferroni-corrected P-Value** |
| --- | --- | --- | --- | --- | --- | --- |
| cg17739917 | -0.069 | 0.007 | 1.782E-21 | 377 | 0.429 | 1.257E-15 |
| cg01940273 | -0.065 | 0.007 | 2.701E-21 | 377 | 0.579 | 1.905E-15 |
| cg21566642 | -0.097 | 0.010 | 1.056E-20 | 377 | 0.535 | 7.447E-15 |
| cg24797066 | -0.021 | 0.002 | 5.163E-20 | 377 | 0.458 | 3.643E-14 |
| cg21161138 | -0.052 | 0.006 | 6.063E-20 | 377 | 0.717 | 4.278E-14 |
| cg05575921 | -0.172 | 0.020 | 2.249E-18 | 377 | 0.810 | 1.587E-12 |
| cg21911711 | -0.033 | 0.004 | 1.187E-16 | 377 | 0.790 | 8.375E-11 |
| cg25648203 | -0.039 | 0.005 | 1.818E-16 | 377 | 0.780 | 1.283E-10 |
| cg14753356 | -0.044 | 0.005 | 1.912E-16 | 377 | 0.460 | 1.349E-10 |
| cg03636183 | -0.058 | 0.007 | 2.351E-16 | 377 | 0.628 | 1.659E-10 |
| cg02738868 | -0.015 | 0.002 | 4.772E-16 | 377 | 0.313 | 3.367E-10 |
| cg18110140 | -0.055 | 0.007 | 2.061E-15 | 377 | 0.492 | 1.454E-09 |
| cg13184736 | -0.054 | 0.007 | 4.911E-15 | 377 | 0.306 | 3.465E-09 |
| cg22675726 | -0.047 | 0.006 | 5.967E-15 | 377 | 0.606 | 4.210E-09 |
| cg21611682 | -0.029 | 0.004 | 1.302E-14 | 377 | 0.580 | 9.186E-09 |
| cg26703534 | -0.043 | 0.006 | 3.512E-14 | 377 | 0.678 | 2.478E-08 |
| cg15159987 | -0.021 | 0.003 | 7.560E-14 | 377 | 0.596 | 5.334E-08 |
| cg10765427 | -0.021 | 0.003 | 1.121E-13 | 377 | 0.545 | 7.909E-08 |
| cg05157376 | -0.034 | 0.005 | 2.049E-13 | 377 | 0.607 | 1.446E-07 |
| cg27537125 | -0.016 | 0.002 | 2.133E-13 | 377 | 0.211 | 1.505E-07 |
| cg07390844 | -0.033 | 0.005 | 9.879E-13 | 377 | 0.476 | 6.970E-07 |
| cg25189904 | -0.068 | 0.010 | 1.013E-12 | 377 | 0.422 | 7.146E-07 |
| cg11902777 | -0.015 | 0.002 | 1.274E-12 | 377 | 0.095 | 8.986E-07 |
| cg07339236 | -0.024 | 0.003 | 2.155E-12 | 377 | 0.157 | 1.520E-06 |
| cg24947681 | -0.026 | 0.004 | 3.716E-12 | 377 | 0.672 | 2.622E-06 |
| cg05086879 | -0.036 | 0.005 | 5.025E-12 | 377 | 0.800 | 3.545E-06 |
| cg05753553 | 0.031 | 0.005 | 1.021E-11 | 377 | 0.684 | 7.204E-06 |
| cg03329539 | -0.033 | 0.005 | 2.684E-11 | 377 | 0.402 | 1.894E-05 |
| cg05009104 | 0.049 | 0.007 | 2.720E-11 | 377 | 0.713 | 1.919E-05 |
| cg22440155 | 0.026 | 0.004 | 2.884E-11 | 377 | 0.484 | 2.035E-05 |
| cg12147622 | -0.027 | 0.004 | 3.815E-11 | 377 | 0.551 | 2.692E-05 |
| cg25748521 | -0.014 | 0.002 | 8.396E-11 | 377 | 0.759 | 5.924E-05 |
| cg00475490 | -0.034 | 0.005 | 9.996E-11 | 377 | 0.180 | 7.052E-05 |
| cg00045592 | -0.036 | 0.006 | 1.452E-10 | 377 | 0.490 | 1.024E-04 |
| cg08709672 | -0.026 | 0.004 | 1.770E-10 | 377 | 0.628 | 1.249E-04 |
| cg24090911 | -0.032 | 0.005 | 2.229E-10 | 377 | 0.748 | 1.573E-04 |
| cg09935388 | -0.074 | 0.012 | 2.422E-10 | 377 | 0.730 | 1.709E-04 |
| cg24859433 | -0.016 | 0.003 | 4.009E-10 | 377 | 0.856 | 2.829E-04 |
| cg06644428 | -0.027 | 0.004 | 4.091E-10 | 377 | 0.098 | 2.886E-04 |
| cg12803068 | 0.084 | 0.014 | 5.336E-10 | 377 | 0.718 | 3.765E-04 |
| cg15885703 | -0.039 | 0.006 | 5.388E-10 | 377 | 0.508 | 3.801E-04 |
| cg09834951 | -0.020 | 0.003 | 7.129E-10 | 377 | 0.434 | 5.030E-04 |
| cg04003530 | 0.039 | 0.006 | 7.841E-10 | 377 | 0.507 | 5.532E-04 |
| cg19572487 | -0.029 | 0.005 | 1.088E-09 | 377 | 0.525 | 7.673E-04 |
| cg00310412 | -0.023 | 0.004 | 1.221E-09 | 377 | 0.540 | 8.615E-04 |
| cg19885130 | -0.036 | 0.006 | 1.475E-09 | 377 | 0.481 | 1.040E-03 |
| cg20295214 | -0.023 | 0.004 | 1.777E-09 | 377 | 0.804 | 1.253E-03 |
| cg09338374 | 0.026 | 0.004 | 1.854E-09 | 377 | 0.563 | 1.308E-03 |
| cg01899089 | -0.023 | 0.004 | 1.856E-09 | 377 | 0.523 | 1.309E-03 |
| cg17419818 | -0.026 | 0.004 | 2.136E-09 | 377 | 0.608 | 1.507E-03 |
| cg07986378 | -0.032 | 0.005 | 2.165E-09 | 377 | 0.626 | 1.527E-03 |
| cg00073090 | -0.020 | 0.003 | 2.182E-09 | 377 | 0.368 | 1.540E-03 |
| cg20328799 | 0.026 | 0.004 | 2.547E-09 | 377 | 0.684 | 1.797E-03 |
| cg06534890 | -0.021 | 0.004 | 3.446E-09 | 377 | 0.505 | 2.431E-03 |
| cg17651613 | -0.045 | 0.008 | 4.519E-09 | 377 | 0.301 | 3.189E-03 |
| cg01765406 | -0.017 | 0.003 | 4.765E-09 | 377 | 0.460 | 3.362E-03 |
| cg09099830 | -0.028 | 0.005 | 5.332E-09 | 377 | 0.527 | 3.762E-03 |
| cg16758086 | 0.023 | 0.004 | 6.953E-09 | 377 | 0.273 | 4.905E-03 |
| cg03507326 | -0.034 | 0.006 | 7.437E-09 | 377 | 0.746 | 5.247E-03 |
| cg09010260 | -0.036 | 0.006 | 7.552E-09 | 377 | 0.641 | 5.328E-03 |
| cg04414766 | 0.062 | 0.011 | 7.909E-09 | 377 | 0.603 | 5.580E-03 |
| cg16145216 | 0.034 | 0.006 | 8.800E-09 | 377 | 0.355 | 6.209E-03 |
| cg07741821 | -0.023 | 0.004 | 9.705E-09 | 377 | 0.729 | 6.847E-03 |
| cg00501876 | -0.018 | 0.003 | 9.940E-09 | 377 | 0.590 | 7.013E-03 |
| cg04885881 | -0.032 | 0.006 | 1.087E-08 | 377 | 0.509 | 7.668E-03 |
| cg01010073 | -0.025 | 0.004 | 1.388E-08 | 377 | 0.448 | 9.796E-03 |
| cg10750182 | -0.016 | 0.003 | 1.480E-08 | 377 | 0.621 | 1.044E-02 |
| cg17936567 | -0.012 | 0.002 | 1.580E-08 | 377 | 0.301 | 1.115E-02 |
| cg26963277 | -0.017 | 0.003 | 1.619E-08 | 377 | 0.891 | 1.142E-02 |
| cg05284742 | -0.018 | 0.003 | 2.112E-08 | 377 | 0.738 | 1.490E-02 |
| cg23583523 | -0.032 | 0.006 | 2.171E-08 | 377 | 0.448 | 1.532E-02 |
| cg15542713 | 0.050 | 0.009 | 2.600E-08 | 377 | 0.456 | 1.835E-02 |
| cg07865653 | -0.020 | 0.004 | 2.723E-08 | 377 | 0.677 | 1.921E-02 |
| cg22441770 | -0.013 | 0.002 | 3.251E-08 | 377 | 0.695 | 2.294E-02 |
| cg26673040 | -0.015 | 0.003 | 3.420E-08 | 377 | 0.826 | 2.413E-02 |
| cg25953130 | -0.032 | 0.006 | 3.549E-08 | 377 | 0.532 | 2.504E-02 |
| cg10420527 | -0.015 | 0.003 | 3.610E-08 | 377 | 0.551 | 2.547E-02 |
| cg22222502 | -0.022 | 0.004 | 3.649E-08 | 377 | 0.827 | 2.574E-02 |
| cg14624207 | -0.017 | 0.003 | 4.177E-08 | 377 | 0.535 | 2.947E-02 |
| cg19089201 | 0.053 | 0.010 | 4.373E-08 | 377 | 0.782 | 3.085E-02 |
| cg27241845 | -0.030 | 0.006 | 4.661E-08 | 377 | 0.664 | 3.289E-02 |
| cg23480021 | 0.059 | 0.011 | 4.665E-08 | 377 | 0.713 | 3.291E-02 |
| cg18625627 | 0.029 | 0.005 | 4.703E-08 | 377 | 0.279 | 3.318E-02 |
| cg16143121 | 0.028 | 0.005 | 5.462E-08 | 377 | 0.553 | 3.854E-02 |
| cg15342087 | -0.013 | 0.002 | 5.639E-08 | 377 | 0.860 | 3.979E-02 |
| cg27519828 | 0.015 | 0.003 | 6.174E-08 | 377 | 0.552 | 4.356E-02 |
| cg00827939 | -0.018 | 0.003 | 6.307E-08 | 377 | 0.739 | 4.450E-02 |
| cg16522223 | -0.014 | 0.003 | 6.323E-08 | 377 | 0.294 | 4.461E-02 |
