# Supplementary material for: Epigenetic markers of disease risk and psychotherapy response in anxiety disorders – a longitudinal analysis of the DNA methylome
Source: Mol Psychiatry. 2025 Apr 25;30(10):4529–42. doi: 10.1038/s41380-025-03038-5 (PMC12436192; doi:10.1038/s41380-025-03038-5)
Supplement: Supplementary file 3 — Supplementary Table 3 [file 41380_2025_3038_MOESM3_ESM.docx]

**Supplementary Table S3:** Gene Ontology Enrichment for genes implicated in the case control analysis (p<0.05).

| **ID** | **Description** | **GeneRatio** | **BgRatio** | **Rich**  **Factor** | **Fold**  **Enrichment** | **zScore** | **pvalue** | **p.adjust** |
| --- | --- | --- | --- | --- | --- | --- | --- | --- |
| GO:0010831 | positive regulation of myotube differentiation | 3/103 | 17/18986 | 0.176 | 32.529 | 9.61 | 9.98E-05 | 0.139 |
| GO:0051149 | positive regulation of muscle cell differentiation | 4/103 | 84/18986 | 0.048 | 8.778 | 5.28 | 1.13E-03 | 0.584 |
| GO:0010830 | regulation of myotube differentiation | 3/103 | 47/18986 | 0.064 | 11.766 | 5.46 | 2.11E-03 | 0.584 |
| GO:0051155 | positive regulation of striated muscle cell differentiation | 3/103 | 51/18986 | 0.059 | 10.843 | 5.20 | 2.67E-03 | 0.584 |
| GO:0071709 | membrane assembly | 3/103 | 63/18986 | 0.048 | 8.778 | 4.57 | 4.86E-03 | 0.584 |
| GO:0070314 | G1 to G0 transition | 2/103 | 20/18986 | 0.100 | 18.433 | 5.76 | 5.20E-03 | 0.584 |
| GO:0044091 | membrane biogenesis | 3/103 | 70/18986 | 0.043 | 7.900 | 4.27 | 6.52E-03 | 0.584 |
| GO:0051443 | positive regulation of ubiquitin-protein transferase activity | 2/103 | 23/18986 | 0.087 | 16.029 | 5.33 | 6.85E-03 | 0.584 |
| GO:0000086 | G2/M transition of mitotic cell cycle | 4/103 | 141/18986 | 0.028 | 5.229 | 3.72 | 7.31E-03 | 0.584 |
| GO:1901880 | negative regulation of protein depolymerization | 3/103 | 74/18986 | 0.041 | 7.473 | 4.12 | 7.61E-03 | 0.584 |
| GO:0043242 | negative regulation of protein-containing complex disassembly | 3/103 | 84/18986 | 0.036 | 6.583 | 3.79 | 1.08E-02 | 0.584 |
| GO:0044839 | cell cycle G2/M phase transition | 4/103 | 158/18986 | 0.025 | 4.667 | 3.42 | 1.08E-02 | 0.584 |
| GO:0007214 | gamma-aminobutyric acid signaling pathway | 2/103 | 30/18986 | 0.067 | 12.289 | 4.57 | 1.15E-02 | 0.584 |
| GO:0099068 | postsynapse assembly | 3/103 | 87/18986 | 0.034 | 6.356 | 3.70 | 1.18E-02 | 0.584 |
| GO:0033673 | negative regulation of kinase activity | 4/103 | 166/18986 | 0.024 | 4.442 | 3.29 | 1.28E-02 | 0.584 |
| GO:0051147 | regulation of muscle cell differentiation | 4/103 | 166/18986 | 0.024 | 4.442 | 3.29 | 1.28E-02 | 0.584 |
| GO:0009584 | detection of visible light | 2/103 | 32/18986 | 0.063 | 11.521 | 4.40 | 1.30E-02 | 0.584 |
| GO:0035767 | endothelial cell chemotaxis | 2/103 | 32/18986 | 0.063 | 11.521 | 4.40 | 1.30E-02 | 0.584 |
| GO:1901879 | regulation of protein depolymerization | 3/103 | 92/18986 | 0.033 | 6.011 | 3.56 | 1.37E-02 | 0.584 |
| GO:0007009 | plasma membrane organization | 4/103 | 173/18986 | 0.023 | 4.262 | 3.18 | 1.46E-02 | 0.584 |
| GO:0007338 | single fertilization | 4/103 | 175/18986 | 0.023 | 4.213 | 3.15 | 1.52E-02 | 0.584 |
| GO:0051153 | regulation of striated muscle cell differentiation | 3/103 | 97/18986 | 0.031 | 5.701 | 3.43 | 1.58E-02 | 0.584 |
| GO:0043086 | negative regulation of catalytic activity | 7/103 | 486/18986 | 0.014 | 2.655 | 2.73 | 1.66E-02 | 0.584 |
| GO:0001953 | negative regulation of cell-matrix adhesion | 2/103 | 40/18986 | 0.050 | 9.217 | 3.84 | 1.99E-02 | 0.584 |
| GO:0051693 | actin filament capping | 2/103 | 40/18986 | 0.050 | 9.217 | 3.84 | 1.99E-02 | 0.584 |
| GO:0051348 | negative regulation of transferase activity | 4/103 | 190/18986 | 0.021 | 3.881 | 2.95 | 1.99E-02 | 0.584 |
| GO:0051438 | regulation of ubiquitin-protein transferase activity | 2/103 | 41/18986 | 0.049 | 8.992 | 3.78 | 2.08E-02 | 0.584 |
| GO:1904706 | negative regulation of vascular associated smooth muscle cell proliferation | 2/103 | 42/18986 | 0.048 | 8.778 | 3.73 | 2.18E-02 | 0.584 |
| GO:0098703 | calcium ion import across plasma membrane | 2/103 | 43/18986 | 0.047 | 8.573 | 3.67 | 2.28E-02 | 0.584 |
| GO:1902656 | calcium ion import into cytosol | 2/103 | 43/18986 | 0.047 | 8.573 | 3.67 | 2.28E-02 | 0.584 |
| GO:0030835 | negative regulation of actin filament depolymerization | 2/103 | 44/18986 | 0.045 | 8.379 | 3.62 | 2.38E-02 | 0.584 |
| GO:0150052 | regulation of postsynapse assembly | 2/103 | 44/18986 | 0.045 | 8.379 | 3.62 | 2.38E-02 | 0.584 |
| GO:0019098 | reproductive behavior | 2/103 | 45/18986 | 0.044 | 8.192 | 3.57 | 2.48E-02 | 0.584 |
| GO:0002062 | chondrocyte differentiation | 3/103 | 116/18986 | 0.026 | 4.767 | 3.01 | 2.53E-02 | 0.584 |
| GO:1903076 | regulation of protein localization to plasma membrane | 3/103 | 117/18986 | 0.026 | 4.726 | 2.99 | 2.58E-02 | 0.584 |
| GO:0140467 | integrated stress response signaling | 2/103 | 48/18986 | 0.042 | 7.680 | 3.42 | 2.80E-02 | 0.584 |
| GO:0007162 | negative regulation of cell adhesion | 5/103 | 314/18986 | 0.016 | 2.935 | 2.55 | 2.82E-02 | 0.584 |
| GO:0051261 | protein depolymerization | 3/103 | 122/18986 | 0.025 | 4.533 | 2.89 | 2.88E-02 | 0.584 |
| GO:0030521 | androgen receptor signaling pathway | 2/103 | 49/18986 | 0.041 | 7.524 | 3.38 | 2.91E-02 | 0.584 |
| GO:0009566 | fertilization | 4/103 | 216/18986 | 0.019 | 3.414 | 2.63 | 3.01E-02 | 0.584 |
| GO:0035384 | thioester biosynthetic process | 2/103 | 50/18986 | 0.040 | 7.373 | 3.33 | 3.02E-02 | 0.584 |
| GO:0071616 | acyl-CoA biosynthetic process | 2/103 | 50/18986 | 0.040 | 7.373 | 3.33 | 3.02E-02 | 0.584 |
| GO:0035036 | sperm-egg recognition | 2/103 | 51/18986 | 0.039 | 7.229 | 3.29 | 3.13E-02 | 0.584 |
| GO:0038084 | vascular endothelial growth factor signaling pathway | 2/103 | 51/18986 | 0.039 | 7.229 | 3.29 | 3.13E-02 | 0.584 |
| GO:0043244 | regulation of protein-containing complex disassembly | 3/103 | 129/18986 | 0.023 | 4.287 | 2.77 | 3.32E-02 | 0.584 |
| GO:0030834 | regulation of actin filament depolymerization | 2/103 | 53/18986 | 0.038 | 6.956 | 3.21 | 3.36E-02 | 0.584 |
| GO:0032330 | regulation of chondrocyte differentiation | 2/103 | 53/18986 | 0.038 | 6.956 | 3.21 | 3.36E-02 | 0.584 |
| GO:0072330 | monocarboxylic acid biosynthetic process | 4/103 | 224/18986 | 0.018 | 3.292 | 2.55 | 3.37E-02 | 0.584 |
| GO:0014902 | myotube differentiation | 3/103 | 133/18986 | 0.023 | 4.158 | 2.70 | 3.58E-02 | 0.584 |
| GO:0045931 | positive regulation of mitotic cell cycle | 3/103 | 136/18986 | 0.022 | 4.066 | 2.65 | 3.79E-02 | 0.584 |
| GO:0030042 | actin filament depolymerization | 2/103 | 58/18986 | 0.034 | 6.356 | 3.02 | 3.96E-02 | 0.584 |
| GO:0043547 | positive regulation of GTPase activity | 3/103 | 140/18986 | 0.021 | 3.950 | 2.59 | 4.07E-02 | 0.584 |
| GO:0061041 | regulation of wound healing | 3/103 | 140/18986 | 0.021 | 3.950 | 2.59 | 4.07E-02 | 0.584 |
| GO:0007015 | actin filament organization | 6/103 | 465/18986 | 0.013 | 2.378 | 2.22 | 4.10E-02 | 0.584 |
| GO:0008643 | carbohydrate transport | 3/103 | 142/18986 | 0.021 | 3.894 | 2.56 | 4.22E-02 | 0.584 |
| GO:0007160 | cell-matrix adhesion | 4/103 | 241/18986 | 0.017 | 3.059 | 2.38 | 4.23E-02 | 0.584 |
| GO:0009581 | detection of external stimulus | 3/103 | 144/18986 | 0.021 | 3.840 | 2.53 | 4.36E-02 | 0.584 |
| GO:0051932 | synaptic transmission, GABAergic | 2/103 | 62/18986 | 0.032 | 5.946 | 2.88 | 4.47E-02 | 0.584 |
| GO:0006470 | protein dephosphorylation | 3/103 | 146/18986 | 0.021 | 3.788 | 2.50 | 4.52E-02 | 0.584 |
| GO:0009582 | detection of abiotic stimulus | 3/103 | 146/18986 | 0.021 | 3.788 | 2.50 | 4.52E-02 | 0.584 |
| GO:0002011 | morphogenesis of an epithelial sheet | 2/103 | 64/18986 | 0.031 | 5.760 | 2.82 | 4.73E-02 | 0.584 |
| GO:0008380 | RNA splicing | 6/103 | 484/18986 | 0.012 | 2.285 | 2.12 | 4.82E-02 | 0.584 |
| GO:0030837 | negative regulation of actin filament polymerization | 2/103 | 65/18986 | 0.031 | 5.672 | 2.79 | 4.86E-02 | 0.584 |
| GO:1903078 | positive regulation of protein localization to plasma membrane | 2/103 | 65/18986 | 0.031 | 5.672 | 2.79 | 4.86E-02 | 0.584 |
| GO:0031333 | negative regulation of protein-containing complex assembly | 3/103 | 152/18986 | 0.020 | 3.638 | 2.41 | 4.98E-02 | 0.584 |
| GO:0010812 | negative regulation of cell-substrate adhesion | 2/103 | 66/18986 | 0.030 | 5.586 | 2.76 | 5.00E-02 | 0.584 |
| GO:0017156 | calcium-ion regulated exocytosis | 2/103 | 66/18986 | 0.030 | 5.586 | 2.76 | 5.00E-02 | 0.584 |
| GO:0045744 | negative regulation of G protein-coupled receptor signaling pathway | 2/103 | 66/18986 | 0.030 | 5.586 | 2.76 | 5.00E-02 | 0.584 |
