# Supplementary material for: Epigenetic markers of disease risk and psychotherapy response in anxiety disorders – a longitudinal analysis of the DNA methylome
Source: Mol Psychiatry. 2025 Apr 25;30(10):4529–42. doi: 10.1038/s41380-025-03038-5 (PMC12436192; doi:10.1038/s41380-025-03038-5)
Supplement: Supplementary file 4 — Supplementary Table 4 [file 41380_2025_3038_MOESM4_ESM.docx]

**Supplementary Table S4:** Epigenome-wide significant CpG-sites associated with treatment response after treatment (POST) and at follow-up (FU). Treatment response was assessed continuously as percentage of HAM-A change.

| Table S4a.  Epigenome-wide significant CpG- sites associated with treatment response at POST (% HAM-A change) | | | | | | | | |
| --- | --- | --- | --- | --- | --- | --- | --- | --- |
| **probeID** | **Chr** | **Position** | **Effect** | **SE** | **P** | **N** | **Bonferroni** | **Gene** |
| cg00856443 | 8 | 60127667 | 319.30 | 54.28 | 4.03E-09 | 335 | 0.0028889 |  |
| cg10322510 | 16 | 12136822 | -45.34 | 5.62 | 7.45E-16 | 334 | 5.33E-10 | *RUNDC2A* |
| cg11851429 | 1 | 110037126 | 92.98 | 14.91 | 4.44E-10 | 335 | 0.00031833 | *CYB561D1* |
| cg19104068 | 13 | 32615885 | 183.31 | 22.61 | 5.24E-16 | 335 | 3.75E-10 | *FRY* |
| cg20423714 | 11 | 19677868 | 192.77 | 32.60 | 3.34E-09 | 335 | 0.00239475 | *NAV2* |
| cg25128170 | 20 | 60425143 | 179.47 | 15.59 | 1.19E-30 | 335 | 8.50E-25 | *CDH4* |
|  |  |  |  |  |  |  |  |  |
|  |  |  |  |  |  |  |  |  |
| Table S4b. Epigenome-wide significant CpG-sites associated with treatment response at FU (% HAM-A change) | | | | | | | | |
| **probeID** | **Chr** | **Position** | **Effect** | **SE** | **P** | **N** | **Bonferroni** | **Gene** |
| cg00037940 | 3 | 138479285 | 165.84 | 25.20 | 4.71E-11 | 306 | 3.34E-05 | *PIK3CB* |
| cg07116649 | 20 | 43741240 | 281.10 | 45.12 | 4.65E-10 | 306 | 0.00032981 | *WFDC5* |
| cg07242808 | 8 | 37476909 | 206.18 | 36.37 | 1.44E-08 | 306 | 0.01020808 |  |
| cg07504272 | 10 | 10952924 | 216.86 | 36.20 | 2.09E-09 | 306 | 0.0014833 |  |
| cg09464883 | 4 | 79689621 | 254.70 | 44.50 | 1.04E-08 | 306 | 0.00741756 |  |
| cg14585004 | 20 | 54874331 | 265.53 | 39.66 | 2.15E-11 | 306 | 1.52E-05 |  |
| cg14628819 | 7 | 105258666 | 213.51 | 31.91 | 2.21E-11 | 306 | 1.57E-05 | *ATXN7L1* |
| cg15377758 | 4 | 1052676 | 222.48 | 40.70 | 4.61E-08 | 306 | 0.03271975 |  |
| cg15849439 | 3 | 37456129 | 202.62 | 23.89 | 2.23E-17 | 306 | 1.58E-11 | *C3orf35* |
| cg22303101 | 5 | 137672952 | 347.51 | 63.06 | 3.57E-08 | 306 | 0.02534428 | *FAM53C;CDC25C* |
| cg23448879 | 12 | 668553 | 283.96 | 35.15 | 6.61E-16 | 306 | 4.69E-10 | *B4GALNT3* |
| cg23820660 | 17 | 78283015 | 223.09 | 41.13 | 5.82E-08 | 306 | 0.04135137 | *RNF213* |
| cg25391562 | 5 | 89806996 | 269.99 | 40.61 | 2.95E-11 | 306 | 2.09E-05 | *POLR3G* |
