# Supplementary material for: Epigenetic markers of disease risk and psychotherapy response in anxiety disorders – a longitudinal analysis of the DNA methylome
Source: Mol Psychiatry. 2025 Apr 25;30(10):4529–42. doi: 10.1038/s41380-025-03038-5 (PMC12436192; doi:10.1038/s41380-025-03038-5)
Supplement: Supplementary file 5 — Supplementary Table 5 [file 41380_2025_3038_MOESM5_ESM.docx]

**Supplementary Table S5:** Overlapping genes implied by CpG sites identified to be significantly associated with anxiety disorders in the present study and as reported in a cross-anxiety disorder, cell type-specific epigenome-wide association study (EWAS) by Hettema et al. [1]

| **Whole Blood** | **T-Cells** | **Monocytes** | **Granulocytes** | **B-Cells** |
| --- | --- | --- | --- | --- |
| *ACSF3* | ***AJAP1*** | ***ATP11A*** | ***AJAP1*** | *AP4S1* |
| ***CTIF*** | ***APPBP2*** | ***CCDC57*** | ***ATF1*** | ***CNOT6*** |
| ***EXD3*** | *C3orf67* | ***CNOT6*** | ***CCDC57*** | ***COL22A1*** |
| ***GALNTL6*** | ***CD27-AS1*** | ***CYHR1*** | ***CNOT6*** | ***CTIF*** |
| ***IQCA1*** | ***EXD3*** | ***EXD3*** | ***CTIF*** | ***FBRSL1*** |
| ***L3MBTL4*** | *GAB1* | ***FBRSL1*** | ***FBRSL1*** | ***GALNTL6*** |
| ***PTPRN2*** | ***GALNTL6*** | ***GDPD3*** | ***GALNTL6*** | ***HIBADH*** |
| ***SIPA1L3*** | ***GNG12-AS1*** | ***GSE1*** | ***GNG12-AS1*** | ***IL12RB2*** |
| ***SMOC2*** | ***GSE1*** | ***KDM4B*** | ***HSPA12A*** | ***KDM4B*** |
| *TAX1BP1* | ***GXYLT2*** | ***L3MBTL4*** | ***IQCA1*** | ***PMEPA1*** |
| ***TRHDE*** | ***MCPH1*** | ***MAML1*** | ***MAML1*** | ***PTPRN2*** |
| *WDR27* | ***PPP1R8*** | ***MYBPC1*** | ***MCPH1*** | ***RIMBP2*** |
| ***WDR7*** | ***PTPRN2*** | *PIEZO1* | ***MYBPC1*** | *USP22* |
| ***ZBTB17*** | ***RGS7*** | ***PTPRN2*** | ***PTPRN2*** | ***ZFAND3*** |
| ***ZFAND3*** | ***RIMBP2*** | ***RGS7*** | ***RGS7*** |  |
|  | ***RPH3AL*** | ***RIMBP2*** | ***RIMBP2*** |  |
|  | *SPTBN1* | ***RPH3AL*** | ***SLC45A4*** |  |
|  | *SYNE2* | ***SLC45A4*** | ***ZFAND3*** |  |
|  | ***WDR7*** | ***SMOC2*** |  |  |
|  | ***ZFAND3*** | *TAF2* |  |  |
|  |  | ***TAOK1**** |  |  |

Legend to Supplementary Tab. S5: Alphabetical order; bold: genes implied in ≥ two cell types in the cross-anxiety disorder phenotype EWAS by Hettema et al. [1]; bold and underlined: genes implied across all cell types in the cross-anxiety disorder phenotype EWAS by Hettema et al. [1]; ***** significantly associated with the cross-anxiety disorder phenotype both in the present study and in Hettema et al. [1].

1. Hettema JM, van den Oord E, Zhao M, Xie LY, Copeland WE, Penninx B et al. Methylome-wide association study of anxiety disorders. *Mol Psychiatry* 2023; **28:** 3484-3492.
