# Supplementary figures and images for: Epigenetic markers of disease risk and psychotherapy response in anxiety disorders – a longitudinal analysis of the DNA methylome
Source: Mol Psychiatry. 2025 Apr 25;30(10):4529–42. doi: 10.1038/s41380-025-03038-5 (PMC12436192; doi:10.1038/s41380-025-03038-5)

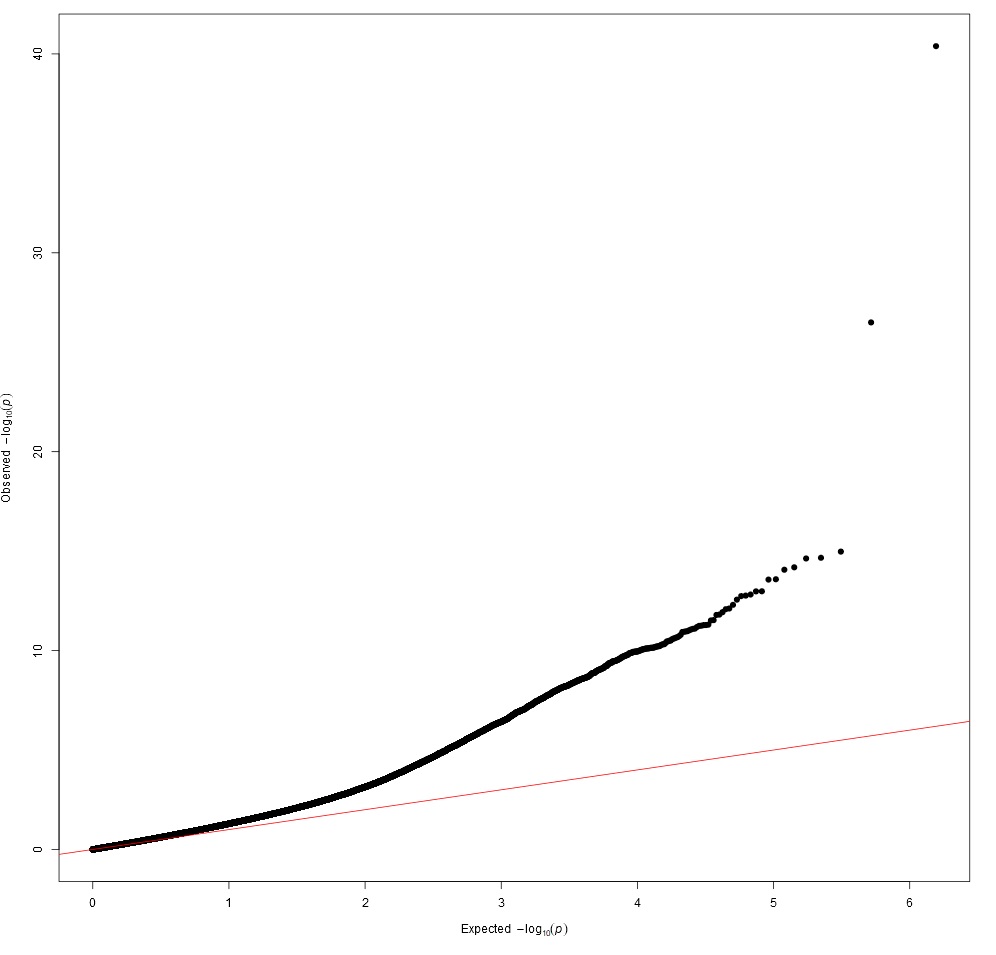

Supplement: Supplementary file 6 — Supplementary Figure 1 [file 41380_2025_3038_MOESM6_ESM.jpg]
