# Supplementary material for: Epigenetic markers of disease risk and psychotherapy response in anxiety disorders – a longitudinal analysis of the DNA methylome
Source: Mol Psychiatry. 2025 Apr 25;30(10):4529–42. doi: 10.1038/s41380-025-03038-5 (PMC12436192; doi:10.1038/s41380-025-03038-5)
Supplement: Supplementary file 7 — Title and legend to Supplementary Figure 1 [file 41380_2025_3038_MOESM7_ESM.docx]

**Supplementary Figure S1:** QQ-Plot for the matched case-control epigenome-wide association study (EWAS) in patients with anxiety disorders (N=378) *vs*. healthy controls (N=295).

Supplementary Fig. S1. Quantile-Quantile (QQ) plot for the case-control EWAS. QQ-plots visualize the deviation of the observed -log10-transformed p-values from the theoretical null distribution (inflation). The inflation factor corresponding to the plot is lambda=1.32856.
